# Geometric morphometrics based diagnostic model for Skeletal Class III patients

**DOI:** 10.1038/s43856-026-01557-y

**Published:** 2026-04-14

**Authors:** Maria Cristina Faria-Teixeira, Inês M. N. Carvalho, Alexandra Dehesa-Santos, Francisco Salvado e Silva, Helena Afonso Agostinho, Francisco Do Vale, António Vaz-Carneiro, Leixuri De Frutos-Valle, Shin-Jae Lee, Joao C. Guimaraes, Alejandro Iglesias-Linares

**Affiliations:** 1 Complutense University of Madrid, School of Dentistry, Madrid, Spain; 2 Universidade de Lisboa, Faculdade de Medicina, Avenida Professor Egas Moniz, 1649-028 Lisboa, Portugal; 3 São João University Hospital of Porto, Porto, Portugal; 4 University of Coimbra, School of Dentistry, Coimbra, Portugal; 5 Institute for Evidence Based Healthcare, Faculdade de Medicina, Universidade de Lisboa, Lisbon, Portugal; 6 Department of Orthodontics, Seoul National University School of Dentistry, 101 Daehak-ro, Jongno-gu, Seoul, Korea; 7 BIOCRAN (Craniofacial Biology) Research Group, Complutense University, 28040 Madrid, Spain

**Keywords:** Dentistry, Computational biology and bioinformatics

## Abstract

**Background:**

Skeletal Class III (SCIII) malocclusion represents a heterogeneous cluster of craniofacial anomalies characterised by a sagittal mesial discrepancy. It is among the most challenging orthodontic conditions to treat because there is no standardisation regarding subphenotype classification or treatment efficacy prediction. This study aimed to develop a data-driven model to identify novel clinically relevant SCIII subphenotypes, contributing to tailored treatment protocols.

**Methods:**

A clinical subphenotypic classification model for SCIII was developed using 12 annotated craniofacial landmarks from lateral cephalometric radiographs of 655 adult SCIII patients of white origin. SCIII subphenotypes were identified by applying generalised Procrustes analysis and unsupervised clustering, and a classification model was developed for predicting subphenotypes for new patients. Cross-validation was employed to demonstrate the robustness of our clustering and classification models.

**Results:**

Here we show that our model inferred six distinct subphenotypes that unravelled relevant morphological features in SCIII patients. We further demonstrate the generalisability of our model across ethnicities using an external validation cohort of patients of Korean origin. The identified SCIII subphenotypes exhibit a strong correlation with treatment decision.

**Conclusions:**

Our results contribute to the development of an accurate SCIII diagnostic tool (available at https://tools.istars.pt/sciii/), moving towards the goal of improving treatment efficacy for this condition.

## Plain Language Summary

Skeletal Class III (SCIII) is a disorder of the jaws representing a major challenge in orthodontics. Diagnosis of SCIII is complex, as patients can often present very differently. Aiming to overcome these limitations, we developed a computer algorithm, based on 2D X-rays of 655 patients, to identify the six most relevant types of SCIII. Our system identified features of patients that allowed for classification of new patients into specific diagnostic groups. The algorithm showed strong ability to predict useful treatment courses and operated well across multiple ethnicities. The presented system will likely prove useful for diagnosing specific subtypes of SCIII, benefiting researchers and clinicians alike.

## Abstract (web summary)

Faria-Teixeira, Carvalho et al. present a machine learning model designed to identify novel diagnostic groups for skeletal Class III malocclusion using cephalometric radiographs from 655 patients. The presented model identifies six distinct subphenotypes that generalise across ethnicities and correlate with treatment selection.

## Introduction

Skeletal Class III (SCIII) phenotype is an umbrella term concerning a multiplicity of phenotypes classically characterised by a sagittal mesial skeletal/dental discrepancy of the lower jaw and a concave soft tissue profile that may occur as an isolated craniofacial trait^1^ or as part of a particular syndrome^2^. SCIII is a multifactorial trait related to genetic and environmental factors that imposes a significant burden on affected individuals regarding their masticatory function, aesthetic appearance, self-esteem and mental health^3–6^. This condition affects growing and non-growing patients^7–9^, with a prevalence of (3.4 ± 1.4) % in Europe, (4.1 ± 1.4) % in America, (4.8 ± 4.2) % in Africa and (7.8 ± 4.2) % in Asia^10–14^.

Two-dimensional (2D) lateral X-rays are considered the primary source for the craniofacial diagnosis of SCIII based on the generated cephalometric quantitative and qualitative phenotypes^15^. Recent studies have suggested multivariate approaches to analyse the complex interactions of cephalometric variables through principal component analysis (PCA) and cluster analysis^10,15^, which have been widely validated across the medical field^16,17^, and have provided insights into SCIII phenotypic variation^8,18–20^. However, these techniques have some limitations, as they rely on distances and angles between anatomical landmarks to categorise patients according to their phenotypes^8,9^. Geometric morphometrics (GM) allows researchers to overcome these limitations by assessing overall craniofacial shape variations to identify group differences^15^. GM is based on the digitised x- and y-coordinates of landmarks, allowing for greater resolution in shape variation analysis of complex structures than traditional cephalometric techniques^15^. An important GM procedure is generalised Procrustes analysis (GPA), which is a superimposition step for the rotation, scaling and translation of data. This step removes any variation that is not related to the shape, as all geometric shapes are scaled to equivalent sizes. Failing to do so may obscure or eliminate discriminative factors^21^.

SCIII diagnosis and treatment have been considered highly complex by medical professionals^22,23^ based on its aetiologic diversity, heterogeneity of phenotypes and unpredictable growth behaviour^8,15,20^. The current clinical SCIII morphological classification is empirically qualitative and does not adequately reflect the craniofacial growth pathways and structures responsible for the development of this complex phenotype^8,15,19^. Treatment approaches for SCIII include growth redirection and modification through dentofacial orthopaedics, dental compensation therapy in slight to mild cases, and orthognathic surgery in moderate to severe cases^24^. Previous studies on white^9,19,25,26^ and Asian^20,27,28^ populations have reported the existence of different subphenotypes in non-growing patients with SCIII; however, their consistency across ethnicities and their connection with preferential treatment remains understudied. We outline, however, that treatment plan selection for SCIII patients is the result of a complex medical diagnosis, encompassing detailed medical history, clinical and functional assessment (e.g., dual bites, cross bites, asymmetries, pseudo-Class III), cephalometric analysis, facial analysis and patients’ expectations management^15,29^. Furthermore, treatment decision in such cases is highly demanding, and with a high range of variability based on medical professionals’ expertise and subjective judgement^30^.

In this study, we employ machine learning to generate a novel clinical subphenotypic classification of SCIII in a broad white adult population of Southern European origin. We confirm that the identified subphenotypes present relevant morphological features and are strongly correlated with treatment selection. We further validate the generalisation properties of our diagnostic model across ethnicities by predicting subphenotypes for a cohort of SCIII patients of Korean origin. Our computational approach can be broadly applied to other clinical morphometric datasets used for patient stratification.

## Methods

### Study design and ethics statement

This cross-sectional study was conducted following the STROBE guidelines and followed the principles of the Declaration of Helsinki. It was approved by the Clinical Research Ethics Committee of the San Carlos Clinical Hospital of Madrid (reference 19/351-E), Clinical Research Ethics Committee of the Hospital de Santa Maria of Lisbon (reference 200/21), Clinical Research Ethics Committee of the Hospital São João in Porto (reference 342/22), the Institutional Review Board for the Protection of Seoul National University School of Dentistry (S-D20220017) and Clinical Research Ethics Committee of the Faculty of Medicine of the University of Coimbra (reference 231/22). Written informed consent was obtained from all participants prior to enrolment in the study.

### Sampling method and eligibility criteria

This study included a Southern European population of Iberian ancestry^31^. Individuals were identified as Portuguese/Spanish if they had at least a two-generation ancestral lineage of Portuguese/Spanish descent, respectively. Eligibility criteria included patients clinically examined in centric relation, at the final stage of maturation (CVMS): IV or V on lateral radiograph imaging^32^, exhibiting a concave profile and ANB angles ≤ 0° or Wits Appraisal ≤ 0 mm (women) or Wits Appraisal ≤ 1 mm (men), thereby including only true skeletal Class III cases. The exclusion criteria were a history of craniofacial trauma, previous orthopaedic or orthodontic treatment, previous history of craniofacial syndromes or non-syndromic craniofacial dysmorphologies, pseudo-Class III malocclusion, missing or poor-quality records and/or failure to sign an informed consent form.

Two external datasets, one including a population of Korean ancestry^33^ and the other of white ancestry^31^ (the latter acquired within a clinical environment), were further included following the previously described inclusion and exclusion criteria.

### Radiological landmarks and cephalometric measurements

Lateral cephalograms were obtained from all participants before orthodontic treatment. These records were standardised, calibrated, digitised and measured using the Dolphin Imaging software (11.2 Dolphin Imaging & Management Solutions, Chatsworth, CA, USA). The landmark coordinates (2D) were annotated by two operators (M.C.F.T. and A.D.S.) who had undergone prior calibration training on a subset of lateral cephalometric radiographs. As described below, an analysis was conducted to assess the reproducibility and reliability of landmark annotations in a subset of patients.

All radiological landmarks and cephalometric measurements used in this study are described in Supplementary Data 1–Table A.1-2, and share their rationale with previous studies^18,26,34^. Twelve anatomic landmark coordinates were included, representing three anatomical planes: cranial base (Nasion [N], Sella [S], Basion [Ba]), maxilla (Posterior Nasal Spine [PNS], Downs A Point [A]) and mandible (Downs B Point [B], Menton [Me], Gonion [Go], Ramus Point [Ramus], Distal Aspect of Condyle [DCo] and Condylion [Co]). Additionally, fifty-three cephalometric measurements were computed, including linear (mm), angular (°), and proportional (%) measurements.

### Reproducibility and reliability of landmark annotation

A sample of 10 randomly selected cephalometric radiographs was independently measured by two experienced operators (M.C.F.T. and A.D.S.) to evaluate inter-rater reliability. For this sample, a generalised Procrustes analysis (GPA), using library shapes from R^35^, was first performed to align the coordinates (without scaling). Then, the consistency in identifying radiological landmarks was assessed using ANOVA^36^ and intraclass correlation coefficients (ICC for a fixed set of 2 judges to rate each landmark)^37^ using the psych library from R^38^.

### Landmarks Procrustes residuals

GPA was performed on two-dimensional annotated facial landmarks with scaling^35^. This method superimposes all landmarks by applying translation, rotation and scaling to minimise shape-unrelated variations, such as size. The landmark differences between each sample/patient and the mean shape, known as the Procrustes residuals, were then calculated. PCA was conducted^18^ to explore and capture the most significant components of SCIII and the patterns of variation in these residuals.

### Cluster analysis

Unsupervised clustering using the k-means algorithm based on Euclidean distances of the previously obtained Procrustes residuals was performed to identify the most homogeneous groups of individuals representing distinct SCIII subphenotypes. We performed k-means clustering for a range of possible clusters (*k* = 1 to 10). Clustering performance was assessed by calculating within-cluster (desired to be as small as possible) and between-cluster (desired to be as large as possible) distances through different metrics, namely the within-cluster sum of squares and average silhouette width^39^. After comparing clustering metrics and the phenotype shapes for each solution, a partition with 6 clusters was selected according to the clinicians’ (A.I.L. and M.C.F.T.) feedback. Finally, this six-cluster solution was compared to other clustering techniques (including agnes, pam and hierarchical clustering) using statistical clustering metrics.

### Characterisation of SCIII subphenotypes

The six formed clusters were characterised based on the number of patients assigned to them and gender distribution within the cohort. Each cluster was represented by a mean shape, which represents the average morphological geometry of the patients in that cluster (or cluster shape). This allowed for a visual understanding of the position and relationships of landmark coordinates, as well as the represented anatomical line. ANOVA was performed to determine whether there were differences in the landmarks and cephalometric measurements among the formed SCIII subphenotypes (clusters). Post-hoc Wilcoxon tests were applied to assess differences between cluster pairs.

### Cluster stability analysis and subphenotype classification of new patients

Stability and reproducibility were assessed through a validation-based approach^40^, casting the problem into a supervised framework to capture how well partitions/clusters are preserved under perturbations to the original dataset. The reference clusters used for performance evaluation were those formed with the entire dataset, which aligns with the absence of known ground truth and the goal of discovering data-driven clusters. The degree of similarity between patient cluster assignments under perturbation to the original dataset and the reference clusters was assessed by maximising the Jaccard coefficient^41^.

We selected a cross-validation approach^42–44^, to provide more confidence in the computed performance metrics, avoiding biases that can occur due to insufficient testing data affecting the effective detection of the data structure and patterns. The data were divided *k* times into fivefolds (*k* = 5), where *k* − 1 folds are used for training the k-means clustering model (with *k* = 6 clusters). The training error, estimated from the cluster assignment match between the ground truth and each training fold, provides insight into the stability and robustness of the clusters by comparing clusters formed with subsets of data to those formed with all data.

The remaining fold was used for assessing the model’s predictability, where clusters were assigned to unseen patients (new patients) through a supervised algorithm. Specifically, as a predictive model, we selected the k-nearest neighbours^45^ (KNN) with 25 neighbours (based on a heuristic previously described^46^), Euclidean distance and rectangular kernel, using the library kknn from R^45^, but further explored other combinations of hyperparameters through a fivefold grid search. The model was fitted using the training subset data with the reference clusters (see above) as the target variable. The testing error measured the performance of predicting the clusters (i.e., subphenotypes) for new patients compared to the cluster assigned to the ground truth dataset.

### Generalisability of SCIII subphenotypes across external datasets

After fitting our subphenotype model on the cohort of 655 white individuals, we evaluated its generalisability by predicting the SCIII subphenotypes of an external cohort comprising 186 SCIII patients of Korean origin and 85 SCIII white patients. For each patient, we computed the Euclidean distance to the centroid—defined as the mean position of all patients within a given cluster in the feature space—of the predicted cluster. These distances were then compared to the distribution of distances to the centroid observed for the original white cohort. Patients whose distances exceeded the 95th percentile of this reference distribution were classified as outliers.

### The relation between SCIII subphenotypes and treatment

A panel of two experienced academic orthodontists (M.C.F.T and A.I.L.) assessed the patient cohort and classified them into “Ortho-Surgical group” and a “Non-Surgical group” according to their clinical expertise and to the “experienced judge grouping method”^47,48^. The criteria for the inclusion of each judge were: (1) more than 10 years of clinical experience in orthodontics; (2) an MS or a PhD degree in orthodontics or experience as a research supervisor or orthodontic postgraduate; and (3) an academic association in the orthodontic field. Then, to assess the relationship between SCIII subphenotypes and clinicians’ treatment suggestions, we analysed the distribution of treatments across clusters using the Chi-square test.

In addition, we conducted an equivalent analysis but using the predicted treatment assignments from the model described by Stellzig-Eisenhauer et al.^48^, which allowed us to further compare the identified SCIII subphenotypes with an alternative method for predicting treatment decisions.

### Statistical analysis

All analyses were conducted using R version 4.4.0 (2024-04-24)^49^. Statistical analyses assumed a Type I Error of 0.05.

## Results

### SCIII Southern European sample characteristics and operator reliability

In total, 655 patients [315 women (48.09%) and 340 men (51.91%)], representing a Southern European cohort of Iberian ancestry^31^, met the inclusion criteria and were selected for our study (Supplementary Data 1–Table B1). All included participants were adults exhibiting a cervical vertebrae maturation stage (CVMS) grades IV or V, with a mean age of (30.08 ± 10.45) years, mean ANB angle of (−3.53 ± 3.58)°, and mean Wits Appraisal of (−9.81 ± 6.85)° (Supplementary Data 1–Table B2).

The cohort was assessed by two experienced operators (M.C.F.T and A.D.S.), and a reliability analysis was conducted to identify any possible operator-related biases. The analysis of variance (ANOVA) results (Supplementary Data 1–Table B3) showed a highly significant effect of landmarks on measurement variance (*p*-value < 2 × 10^−16^); however, no significant effect of the operator (*p*-value = 1.00) or the interaction between landmark and operator was observed (*p*-value = 0.968). Furthermore, the estimated intraclass correlation coefficient (ICC) values for the annotated landmarks were generally greater than 0.70 (Supplementary Data 1–Table B4), indicating strong reliability across operators.

### GPA for SCIII patient landmarks

**Fig. 1:**
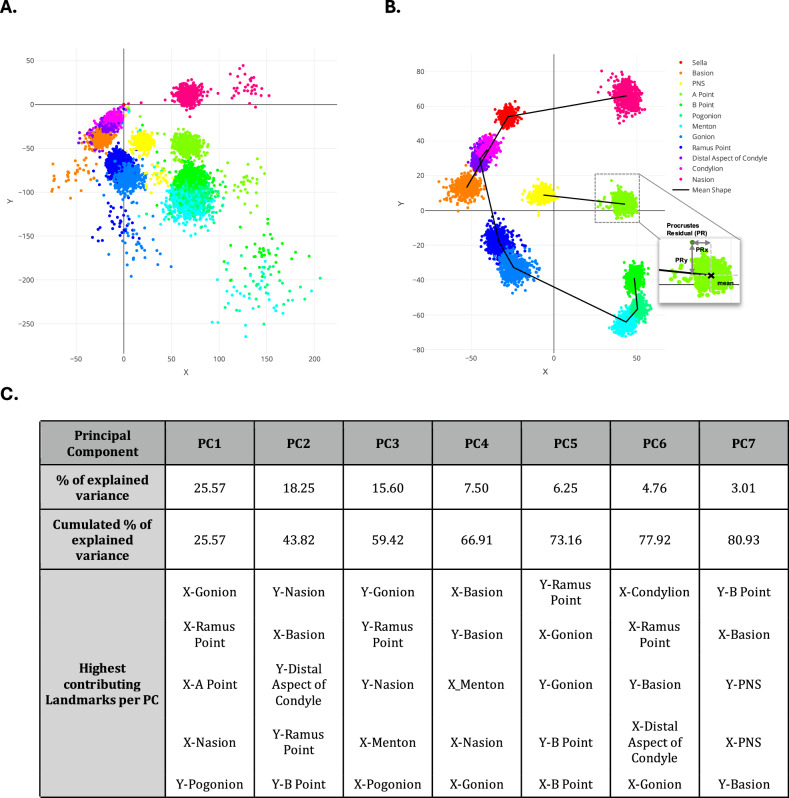
GPA and PCA analysis of SCIII patients. Patients’ landmark coordinates before (**A**) and after (**B**) GPA. The colours represent different patient landmarks. The inset depicts how Procrustes residuals are computed as deviations in the x (PRx) and y (PRy) dimensions from the mean landmark coordinates (black cross). **C** PCA table summary showing explained variance, cumulative explained variance, and the top contributing landmarks for the first seven principal components.

Generalised Procrustes analysis (GPA) enabled the alignment of the 12 landmark coordinates of all the patients and the definition of a corresponding mean shape (Fig. 1A, B and Supplementary Data 1–Table C1). Procrustes residuals were computed for each patient, revealing deviations in their landmark coordinates from the mean shape (Fig. 1B inset, and Supplementary Data 1–Table C2). The principal component analysis (PCA) of Procrustes residuals further showed that seven principal components (PC) explained 80.93% of the total cohort variance (Fig. 1C). The first three PCs, which explained most of the variability across patients (59.42%), were driven by a few landmarks, including [Go], [Ramus], [A], [N] and [DCo]. Deviations in these landmarks are relevant in SCIII patients because they suggest craniofacial deviations during growth and development on both the vertical and sagittal planes and are involved in the severity of the SCIII subphenotypes.

### Unsupervised learning-guided identification of SCIII subphenotypes

**Fig. 2:**
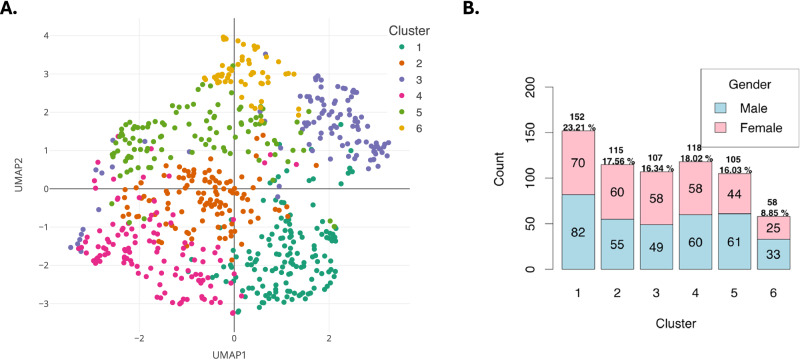
SCIII patients clusters/subphenotypes. **A** UMAP (Uniform Manifold Approximation and Projection) representation of the Procrustes coordinates residuals for the different patients, with the assigned cluster represented by the different colours; **B** size and gender distribution per cluster.

We identified patient clusters with similar skeletal landmark characteristics (or subphenotypes) using an unsupervised learning approach based on the k-means algorithm^50^. To determine the optimal number of clusters, we calculated the within-cluster sum of squares and the silhouette score for a varied number of clusters (*k* = 1 to 10) (Supplementary Data 1–Fig. D1) and found that the former decreased monotonically with an increasing number of clusters, whereas the latter peaked at two clusters and was fairly consistent between three and six clusters, after which there was a sizable drop in the silhouette score. Based on a compromise between clinical applicability and clustering metrics, we selected a solution with six clusters and further compared the clustering performance of k-means with that of other algorithms (Supplementary Data 1–Fig. D2). Our results indicated that the k-means algorithm based on Euclidean distance exhibits a balanced performance across the different metrics, while being substantially better in the identification of more evenly sized clusters (Supplementary Data 1–Fig. D2C), and therefore we selected this solution for further investigation (Fig. 2A, B). Cluster 1 was the largest cluster (*n* = 152, 23.21%), and the smallest was Cluster 6 (*n* = 58, 8.85%). Clusters 2 (*n* = 115, 17.56%), 3 (*n* = 107, 16.34%), 4 (*n* = 118, 18.02%) and 5 (*n* = 105, 16.03%) had comparable sizes. Sex distribution was relatively homogeneous across different clusters (Fig. 2B and Supplementary Data 1–Fig. D3).

### Morphological characteristics of defined clusters

**Fig. 3:**
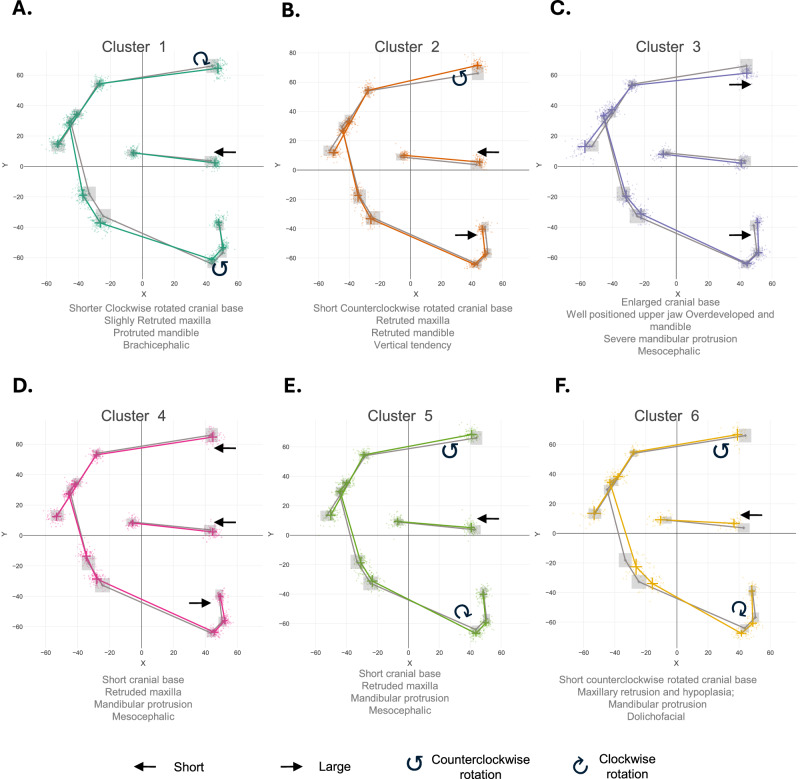
Shape profiles of SCIII patient subphenotypes. **A**–**F** Cluster shape (coloured lines) and respective coordinates mean and standard deviation (coloured error bars) per landmark. Coloured dots around the different landmarks represent measurements for individual patients belonging to each cluster. For reference, the mean shape (grey lines) and respective coordinates mean, and standard deviation (grey boxes) are presented for the whole population.

A geometric morphometrics (GM) analysis further defined the average craniofacial shape for each cluster of SCIII patients (Fig. 3), for which key variables and detailed characteristics are provided in Table 1, Supplementary Data 1–Table D1–D3 and Supplementary Data 1–Fig. D4–D7.

**Table 1:** Summary of the most relevant morphological characteristics, landmarks coordinates and cephalometric variables per cluster

			Cluster 1	Cluster 2	Cluster 3	Cluster 4	Cluster 5	Cluster 6
Main morphological characteristics			Mild SCIII; Shorter Clockwise rotated cranial base; Slighly Retruted maxilla; Protruded mandible; Brachicephalic	Slight SCIII; Short Counterclockwise rotated cranial base; Retruded maxilla; Retruded mandible; Vertical tendency	Severe SCIII; Enlarged cranial base; Well-positioned upper jaw. Overdeveloped, well-positioned mandible; Severe mandibular protrusion; Mesocephalic	Borderline SCIII; Short cranial base; Retruded maxilla; Mandibular protrusion; Mesocephalic	Severe SCIII; Short counterclockwise rotated cranial base; Maxillary retrusion; Mandibular protrusion; Dolichofacial	Severe SCIII; Short counterclockwise rotated cranial base; Maxillary retrusion and hypoplasia; Mandibular protrusion; Dolichofacial
Landmarks	*x*-axis	Positive shift	[S]; [A]; [N]	[Ba]; [A]; [PNS]	[B]	[Pg]; [Me]; [A]	[Ba] [Rasmus]	[Ramus]; [Go]; [Co]; [DCo]
		Negative shift	[Co]; [Go]; [Ramus]; [DCo]	[Me]; [Pg]; [B]	[Ba]; [PNS]	[Ramus]; [Go]; [Co]	[S]; [N]; [A]	[PNS]; [A]; [Me]; [N]; [Pg]
	*y*-axis	Positive shift	[Pg]; [Me]; [Ba]; [B]	[N]; [PNS]	[B]; [DCo]; [Co]	[Go]; [Ramus]	[N]	[DCo]; [Co]; [S]; [A]
		Negative shift	[Co]; [Go]; [A]	[Ba]; [B]; [Co]; [DCo]	[A]; [S]; [Na]; [PNS]	[S]; [PNS]; [A]; [B]	[B]; [Me]; [Pg]	[Pg]; [Me]; [Ramus]
Anatomical structures	Cranial Base		↓Anterior cranial base length (SN) clockwise rotated (↑↑↑SN-Ar)	↓↓↓SN counterclockwise rotated (↑↑SN-Ar)	↑↑↑SN and ↑Posterior cranial base length (S-Ar)	↓↓SN and ↓↓S-Ar	↓↓SN and ↓↓↓Cranial base angle (SN-Ar)	↓↓↓S-Ar and ↓↓SN-Ar
	Maxilla		↑Maxillary and midface lengths ( ↑ ANS-PNS; ↑↑Co-A) Retruded maxilla (↓↓A-NPo; ↑↑SNA; ↑A-Na perp)	Maxillary retrusion (↓↓SNA; ↓A-Na perp) Decreased midface length (↓↓↓Co-A)	↑↑↑Maxillary and midface lengths (↑↑↑Co-A; ↑↑↑ANS-PNS) Well-positioned upper jaw ( ↑ SNA)	↑Maxillary hypoplasia with ↓midface length ( ↓ Co-A) Retruded maxilla (↑↑↑SNA; ↑↑↑A-Na perp)	↑Maxillary retrusion and hypoplasia ↓↓SNA and A-Na perp	↑↑↑Maxillary retrusion and hypoplasia (↓↓↓SNA; ↓↓↓A Na-perp)
	Mandible		↑Mandibular length (Co-Gn) ↓↓Mandibular body length (Go-Gn) Steep mandibular plane (↓↓↓SN GoGn) Mandibular protrusion (↑↑N-A-Pg)	↓↓Mandibular body length (↓↓Go-Gn) Mandibular retrusion (↓↓↓SND; ↓↓↓SNB)	↑↑↑Mandibular length (Co-Gn) ↑↑↑Mandibular body length- (Go-Gn) ↑↑↑Mandibular ramus height (Ar-Go) ↑↑↑Mandibular protrusion (↑↑↑SNB; ↑↑↑SND)	↑Mandibular body length ( ↑ Go-Gn) Horizontal mandibular plane ( ↑ SNGo-Gn) ↓Mandibular ramus height ↑Mandibular protrusion ( ↑ SNB)	↑Mandibular protrusion ↑↑Mandibular length (↑↑ Co-Gn) ↑↑Mandibular body length (↑↑Go-Gn) Clockwise rotation of the mandibular plane (↑↑Sn-GoGn)	↑↑↑Gonial angle
Intermaxillary relation			↑↑ANB ↑↑↑Wits Appraisal ↓↓↓Mx/Md diff (Co-Gn - Co-A)	↑↑↑ ANB ↑↑Wits Appraisal ↓↓Co-Gn - Co-A	↓↓↓ANB ↓↓Wits Appraisal ↑↑Co-Gn - Co-A	↓ANB ↓Wits Appraisal ↓Co-Gn - Co-A	↓ANB ↓Wits Appraisal ↑Co-Gn - Co-A	↓↓ANB ↓↓↓Wits Appraisal ↑↑↑Co-Gn - Co-A
Vertical parameters			↓↓*y*-axis ↓↓↓Lower face height (Ans-Me) ↓↓Anterior face height (NaMe) ↓↓Upper face height (N-ANS) ↓↓↓Gonial angle ↓↓↓Lower Gonial Angle	↑↑↑*y*-axis ↑↑Lower face height (ANS-Xi-Pm) ↑Lower Gonial Angle	↑ANS-Me ↑↑↑Posterior face height (SGo) ↑NaMe ↓Lower Gonial Angle	↓↓*y*-axis ↓↓↓Sgo ↓↓↓NaMe ↑↑↑FMA–Mesocephalic pattern ↓↓Lower Gonial Angle	↑ANS-Xi-Pm ↑↑NaMe ↑↑Articular angle	↑↑↑ANS-Me ↑↑↑NaMe ↑↑↑Gonial Angle (Ar-Go-Me) ↑↑↑Lower Gonial Angle (Na-Go-Me)

↑↑↑Highest parameter; ↑↑Second highest parameter; ↑Increased parameter in the present cohort; ↓↓↓Smallest parameter; ↓↓Second smallest parameter; ↓Diminished parameter in the present cohort.

Cluster 2 (C2 and Fig. 3B) was the less severe SCIII subphenotype with patients exhibiting a short anterior cranial base clockwise rotated [(SN = (68.15 ± 8.79) mm], a retruded maxilla [SNA = (77.93 ± 3.43)°; A-NaPerp = (−0.87 ± 3.38) mm; Co-A = (83.02 ± 11.32) mm] a retruded mandible [SND = (76.06 ± 3.23)°, SNB = (78.38 ± 3.43)°, N-A-Pg = (−2.14 ± 4.88)°], associated to the negative shift of the landmarks [B], [Pg] and [Me] on the horizontal axis (Supplementary Data 1–Table D2 and D3). The lower facial height was increased [ANS-Xi-Pm = (47.68 ± 5.83) mm], contributing to a vertical tendency pattern (Supplementary Data 1–Table D3). Thus, C2 represents a non-surgical slight SCIII phenotype with a vertical tendency related to a shorter counterclockwise rotated cranial base with a retruded maxilla and a retruded mandible.

Cluster 1 (C1 and Fig. 3A) was the second least severe subphenotype. Patients included in C1 exhibited a clockwise-rotated cranial base [SN-Ar = (126.03 ± 5.14)°] and a slightly retruded upper jaw in the sagittal plane [convexity = A-NPo = (−3.47 ± 2.81) mm; SNA = (80.57 ± 3.75)°; A-Na Perp = (0.57 ± 3.16) mm]. Landmarks Y-[Pg], Y-[Me], Y-[Ba] and Y-[B] shifted positively, contributing to a mild protrusion of the mandible [SNB = (82.61 ± 4.16)°; N-A-Pg = (−7.09 ± 5.32)°] with counterclockwise rotation [SN-GoGn = (24.42 ± 4.7)°]. In the vertical plane, the antero-inferior face height measurements were decreased (Supplementary Data 1–Table D3). Overall, C1 represents a brachycephalic non-surgical SCIII mild phenotype with a short clockwise-rotated cranial base, a slightly retruded upper jaw and a protruded mandible.

Cluster 4 (C4 and Fig. 3D), was the third least severe cluster exhibiting a negative shift of Y-[S], resulting in a shorter cranial base length [S-Ar = (32.82 ± 4.27) mm; SN = (68.63 ± 5.65) mm], a reduced midface length [Co-A = (85.21 ± 7.45) mm] and maxillary retrusion [A-NPo = (−3.64 ± 2.64) mm]. C4 patients also exhibited mandibular protrusion [SNB = (83.29 ± 3.45)°; N-A-Pg = (−7.43 ± 5.32)°]. In the vertical plane, Y-[Go] and Y-[Ramus] were shifted positively. The posterior [SGo = (74.53 ± 7.26) mm], anterior [NaMe = (119.08±9.59) mm] and lower face heights [ANS-Xi-Pm = (43.53 ± 4.24) mm] were also decreased (Supplementary Data 1–Table D3). Overall, C4 subphenotype was a mesocephalic phenotype characterised by a shorter anterior and posterior cranial base, a retruded hypoplastic maxilla, and mandibular prognathism, representing a borderline cluster with intermediate characteristics between Clusters 1 and 3 (see below).

The most severe subphenotypes were identified in Clusters 5, 3 and 6. To this respect, cluster 5 (C5 and Fig. 3E) patients had the landmark X-[Ba] shifted positively, and X-[S] and X-[N] shifted very negatively, producing a shorter anterior cranial base [SN = (71.76 ± 17.83) mm] and the lowest mean cranial base angle registered on this cohort [SN-Ar = (122.64 ± 6.31)°]. The maxilla assumed a severely retruded position [SNA = (78.55 ± 4.07)°; A-NA Perp = (−1.09 ± 5.85) mm] and was related to the negative shift of the landmarks X-[N] and X-[A]. The mandible assumed a protruded position with clockwise rotation. In the vertical plane, Y-[B], Y-[Me] and Y-[Pg] shifted negatively, further contributing to the vertical tendency of this phenotype (Supplementary Data 1–Table D2 and D3). Overall, C5 patients exhibited a subphenotype characterised by a shorter counterclockwise-rotated cranial base, mandibular protrusion and severe maxillary retrusion, representing a borderline dolichofacial SCIII phenotype.

The second most severe cluster was cluster 3 (C3 and Fig. 3C). In the horizontal plane X-[N], X-[S], X-[N] and X-[PNS] were shifted negatively, contributing to an increased cranial base length [SN = (78.25 ± 25.85) mm; SGo = (88.03 ± 31.72) mm], and to a well-positioned upper jaw in the sagittal plane [SNA = (80.66 ± 3.55)°; A-Na Perp = (0.53 ± 5.13) mm]. The landmarks position shifted positively for XY-[B], Y-[DCo] and Y-[Co], producing the highest mandibular protrusion of this cohort [SNB = (87.5 ± 3.89)°; SND = (83.98 ± 4.24)°]. In the vertical plane, C3 patients further exhibited a mesofacial pattern with a vertical tendency. Overall, patients included in C3 were mainly characterised by a mesofacial pattern, a well-positioned upper jaw and mandibular protrusion.

Finally, Cluster 6 (C6 and Fig. 3F) patients exhibited landmarks X-[Ramus], X-[Go], X-[Co] and X-[DCo] shifted very positively, causing multiple variables for mandibular dimensions to achieve the highest values in the present cohort (Supplementary Data 1–Table D3). C6 patients exhibited a very negative shift of landmarks X-[N], X-[PNS], X-[A], resulting in the most severe maxillary retrusion observed in this cohort [SNA = (77.76 ± 4.32)°; A-Na Perp = (−1.76 ± 6.84) mm] (Supplementary Data 1–Table D2 and D3). Furthermore, C6 had the shortest mean posterior cranial base [S-Ar = (32.05 ± 8.61) mm] and the second smallest cranial base angle [SN-Ar = (123.61 ± 5.12)°]. The morphometric contrasts observed in the vertical plane contributed to the highest measurements of the lower face height [ANS-Me = (81.97 ± 21.41) mm], anterior face height [NaMe = (136.84 ± 32.77) mm], and gonial angles [Ar-Go-Me = (136.26 ± 8.66)°] of the sample. C6 was considered an extreme phenotype characterised by a reduced posterior cranial base, a hypoplastic and hyperdivergent maxilla, and an overdeveloped prognathic mandible with a dolichofacial pattern.

### Clustering stability and classification accuracy of subphenotypes for new patients

**Fig. 4:**
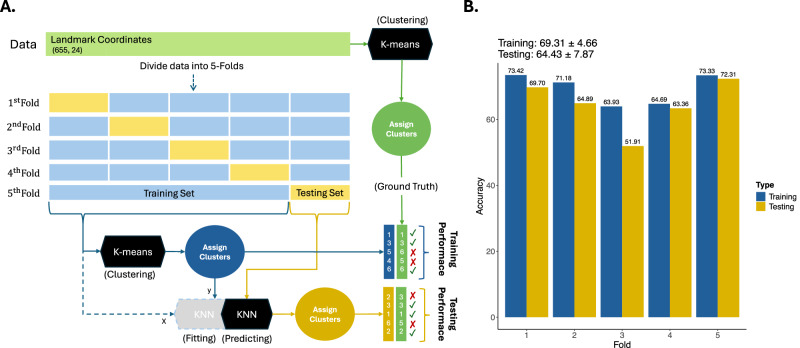
Cluster stability and classification performance. **A** Schematic of the cross-validation method applied to assess model performance, where the training accuracy reflects the cluster-assignment stability, and the testing accuracy quantifies the model’s ability to predict clusters for new patients; **B** training and testing accuracies obtained for each cross-validation fold.

After assessing the clinical relevance of the skeletal features identified for the six clusters, we quantified the stability of patient assignment to the different clusters, as well as the classification accuracy for new patients. For this, we developed a five-fold cross-validation procedure, where each fold took 80% of the patients to perform patient–cluster assignment based on the k-means algorithm, comparing it with the assignment performed when all patients were available (i.e. ground truth) (see Methods and Fig. 4A). The observed performance score (training accuracy) was (69.31 ± 4.66)% (Fig. 4B), suggesting reasonable reliability for the assignment of patient-cluster pairs, even when substantially smaller population sizes were used to define the six distinct clusters. Furthermore, we observed that patients assigned to the wrong cluster exhibited a greater distance to the cluster centre than those that were correctly classified (Supplementary Data 1–Fig. E2), suggesting that misclassified patients might not be core members of the clusters to which they were assigned in the ground truth dataset but may instead represent borderline cases.

We then quantified the classification accuracy of assigning new patients to one of the six previously defined clusters. For this, we used the fivefold cross-validation procedure presented previously, where after defining the six clusters using 80% of the patients, we trained a k-nearest neighbour (KNN) algorithm to predict the cluster for new patients (i.e., the remaining 20% of patients that were left out of the training step) (see Methods and Fig. 4A). Our results showed that the model performance for predicting the cluster for new patients (testing accuracy) was (64.13 ± 7.48)% (Fig. 4B). This performance closely matched the training accuracy, highlighting the ability of the model to correctly assign clusters to unseen patients. We also observed that classification performance was not sensitive to different KNN hypermeters—namely the number of neighbours (k), distance definition and kernel—(Dunn’s post hoc test for Kruskal–Wallis, all *P*-values > 0.05) (Supplementary Data 1—Fig E1).

### SCIII subphenotypes are generalisable to SCIII patients of other ethnicities

**Fig. 5:**
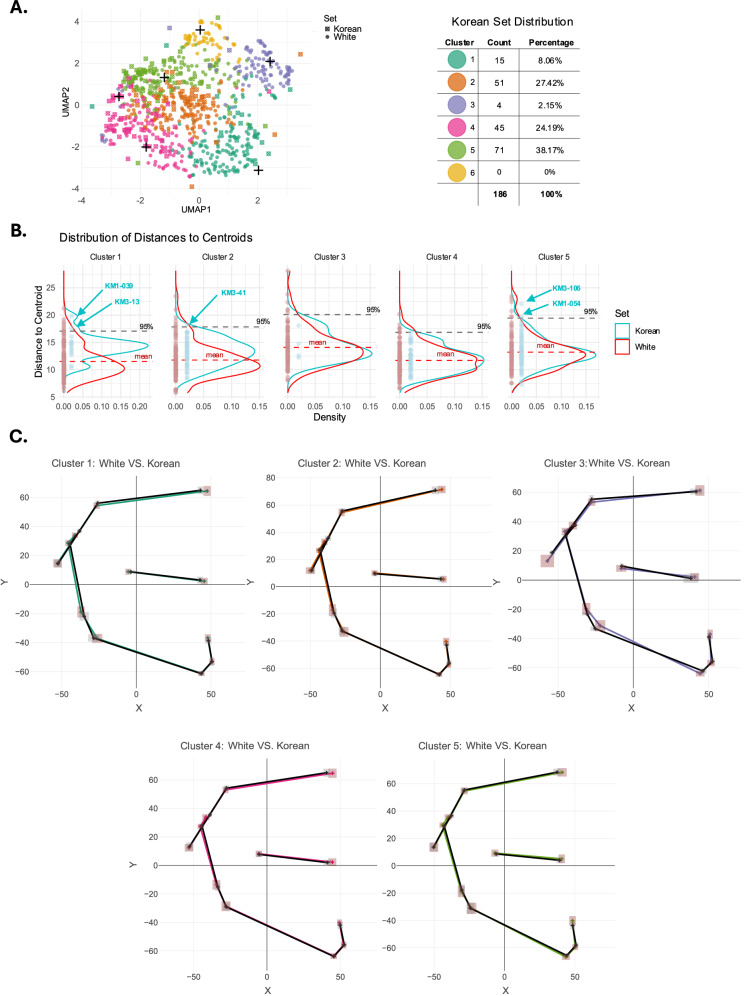
External validation of SCIII subphenotypes – classification in Korean patients. **A** UMAP projection of both training white and external Korean datasets (655 and 186 patients, respectively), along with the assigned cluster/subphenotype (represented by the different colours) (left). Black crosses indicate the centroids of each cluster. Table with the distribution of patients in the external Korean dataset across the six predefined clusters (right). **B** Distance-to-centroid distribution analysis comparing Korean (cyan) and white (red) populations, with identification of potential outliers (exhibiting a distance larger than the 95th percentile observed for the training white distribution, black dashed lines): KM3-13 and KM1-039 in Cluster 1; KM3-41 in Cluster 2; KM3-106 and KM1-054 in Cluster 5. **C** Morphological comparison using the mean shape of white patients (coloured) versus the Korean mean shape (black), by cluster.

After fitting the subphenotype classification model on the 655 patients from the white population, we predicted the subphenotypes for an external dataset composed of 186 SCIII patients of Korean origin (Fig. 5A). The distribution of patients across clusters was as follows: C1: 15, C2: 51, C3: 4, C4: 45, C5: 71 and C6: 0 patients. For each patient, we calculated the distance to the centroid of the assigned cluster, defined as the Euclidean distance between the patient’s morphometric coordinates and the average coordinates of all patients in that cluster (see “Methods”). Most patients fall within the centroid distance distribution observed for the white population (Fig. 5B), and the strong overlap between the mean shapes of the Korean and white populations suggests relevant morphological similarities (Fig. 5C). Nonetheless, five patients were identified as outliers in Clusters 1, 2 and 5 (Supplementary Data 1–Fig. F1).

In contrast, when predicting subphenotypes for an external white patient cohort (*n* = 85), all six subphenotypes were represented (Supplementary Data 1–Fig. F2); however, the distribution differed from that observed in the white dataset used for model training. This discrepancy is expected, given that the external samples originated from a different source (see Methods). In this external white population, Clusters 2 and 4 were the least populated, and a few outliers were also identified (Supplementary Data 1–Fig. F3).

### SCIII subphenotypes are associated with corrective treatment choice

To evaluate the clinical relevance of the identified SCIII clusters, two experienced academic orthodontists (M.C.F.T. and A.I.L.) selected the most adequate corrective treatment for each patient, determining whether surgery was recommended. As illustrated in Table 2, the distribution of treatment assignments in most clusters was significantly different from that expected by chance, as assessed using the Chi-square test, suggesting a strong association between most clusters and specific treatment types (Supplementary Data 1–Fig. G1). Clusters 3, 5 and 6 were more frequently associated with craniofacial surgical treatment, whereas clusters 1 and 2 showed a greater association with non-surgical treatment. Importantly, equivalent relationships between clusters and treatment decision were found when using the treatment suggested by the Stellzig-Eisenhauer et al. model^48^ (Supplementary Data 1–Fig. G2), further asserting the clinical relevance of the SCIII subphenotypes identified, independently of the method used for treatment assignment.

**Table 2:** Distribution of non-surgical and surgical per cluster

Cluster	Total (*n*, %)	Age (years)	Treatment		Ratio surgical/ non-surgical	Chi-Squared test (*p*-value)
			Non-surgical	Surgical		
1	152 (23.20%)	30.68 ± 9.70	119	33	0.28	3.45 × 10^−21^
2	115 (17.56%)	32.81 ± 11.29	88	27	0.31	7.08 × 10^−14^
3	107 (16.34%)	28.24 ± 11.29	6	101	16.83	1.94 × 10^−18^
4	118 (18.02%)	28.49 ± 11.36	54	64	1.19	0.85
5	105 (16.03%)	29.95 ± 10.40	25	80	3.20	4.98 × 10^−06^
6	58 (8.85%)	30.11 ± 9.46	0	58	inf	2.28 × 10^−12^
	655 (100%)	30.08 ± 10.45	292	363		

## Discussion

Previous research has suggested that the number of SCIII subphenotypes could be highly variable due to the heterogeneity of skeletal malocclusion, age, sexual dimorphism, sample size, or ethnic background^9,19,20,25,27,51^. The methodologies used across the up-to-date literature also vary, as some have used hierarchical cluster analysis^25^, whereas others have used mixed cluster analysis^26,52^. Nonetheless, the main limitation of these studies was the use of cephalometric variables, which introduced a dependency on distances and angles to categorise patients according to their subphenotypes. Furthermore, the grouping of cephalometric variables into principal components typically reflects anteroposterior and vertical dimensions rather than craniofacial structures. Thus, there is uncertainty regarding the extent to which the results of previous studies are reliable and generalisable to other populations^27^.

To overcome the limitations of previous studies, we present a machine-learning approach based on GM. We used GM and GPA^21^ to assess craniofacial shape variation by defining the central tendency of the craniofacial shape and computing deviations for individual patients^26^. The adopted methodology allowed for the development of an unsupervised clustering model designed for a broad white adult Iberian population that revealed six different SCIII subphenotypes. Most patients affected by SCIII (58,79%) were included in clusters 1, 2 and 4, which represented slight to moderate maxillary–mandibular discrepancies in the vertical and sagittal planes.

Cluster 2 corresponds to a slight SCIII subphenotype encompassing a vertical tendency related to a short anterior counterclockwise-rotated cranial base with a retruded maxilla and retruded mandible, which has been described previously by our research group^52^. C2 is the third-largest cluster in our external Korean cohort. Our results contrast with previous studies in Asian populations that have not reported this phenotype in detail, highlighting the heterogeneity across different studies and populations^27,53^.

In Cluster 1, the largest cluster of this cohort, patients exhibited a mild SCIII subphenotype, brachycephalic, with a shorter clockwise-rotated cranial base, slight maxillary retrusion, and a protruded mandible. These characteristics were similar to those found in a previous study on a white population, where the authors identified an SCIII subphenotype with slight maxillary retrognathism and mandibular prognathism with a flat mandibular plane^15^. In contrast, Korean population studies reported SCIII subphenotypes where the largest clusters are related to a normal maxilla, hyperdivergent pattern and moderately protrusive mandible, or to a normal maxilla, normodivergent pattern and severely protrusive mandible^54^.

Cluster 4 was considered a borderline mild SCIII subphenotype, with a mean shape exhibiting a mesocephalic, shorter cranial base, a slightly retruded upper jaw, and mandibular protrusion. The characteristics of this cluster were similar to those found in a previous study^52^ in which patients exhibited a mixed maxillo-mandibular origin associated with slight maxillary retrusion and a protruded mandible. This subphenotype exhibits a mesocephalic pattern but a diminished total mandibular size^52^. Interestingly, C4 shares its phenotypic characteristics with an SCIII subphenotype found in a study of patients of Chinese origin^27^. We also found that C4 was well represented on our external cohort of Korean origin (*n* = 45), highlighting the ability of our model to generalise to other ethnic groups.

Clusters 3 and 5 represented severe SCIII subphenotypes, with the C5 mean shape showing a dolichofacial, related to a shorter and counterclockwise-rotated cranial base, severe maxillary retrusion and mandibular protrusion, whereas the C3 mean shape encompassed a mesocephalic, enlarged cranial base with a well-positioned upper jaw, and severe mandibular protrusion. The C3 characteristics matched the smallest cluster reported in a study developed in surgical SCIII patients of Korean origin (normal maxilla, hypodivergent pattern, and severely protrusive mandible)^54^, and accordingly, we found that only 4 patients in our external cohort of Korean origin mapped to this cluster.

Finally, Cluster 6 included patients with an extreme SCIII subphenotype (dolicocephalic) related to a reduced counterclockwise rotation of the cranial base, severe maxillary retrusion and mild mandibular prognathism. The characteristics of C6 were similar to those found in a previous study by our research team^52^, with patients exhibiting the smallest maxillary size, most overdeveloped mandibles, and the highest anterior facial height from all four clusters. Interestingly, there is no previous description of this subphenotype in Chinese populations^27^, and we also could not find any patient belonging to this cluster (*n* = 0) in our external Korean dataset. Notwithstanding, studies in Asian populations describe different extreme subphenotypes (e.g. maxillary retrusion combined with severe mandibular prognathism with a very low plane angle)^52,53^, which were not found in the present research, illustrating the heterogeneity related to the SCIII malocclusion^53^.

The methodology employed in this study offers three important innovations. First, we challenge current approaches relying on cephalometric measurements to identify SCIII subphenotypes and propose that a framework based on GM and machine learning should be used instead. Second, we propose the use of a cross-validation procedure to assess the robustness of subphenotype classification and show that patient grouping from smaller sample sizes is still moderately accurate (~69%), with misclassifications being mostly explained by borderline cases where the patient’s sub-phenotype might not be unique. Our methodology provides a way to identify such cases and contributes to raising awareness on SCIII subphenotypes heterogeneity around scientists and clinicians. We expect that with more patient data becoming available, the definition of SCIII subphenotypes, as proposed by our work, will evolve, and subphenotypes characteristics, as well as borders between clusters, will become more well defined, leading to an overall improved diagnostic accuracy. Third, we present a classification model for accurately assigning new patients to one of six distinct SCIII subphenotypes directly from their morphometric data and validate this approach using two external cohorts of SCIII patients of white and Korean origin. It should be considered, however, that the accuracy of data-driven diagnostic tools is inherently influenced by the reliability of landmark annotations across different operators. In our dataset, most landmark annotations were highly reproducible across two operators, and we verified that patient assignment to subphenotype probabilities was also consistent (average and median cosine similarity of 0.8209 and 0.9660, respectively). Our results highlight the generalisation properties of our diagnostic model, which provides a practical machine learning-based tool for supporting the clinical diagnosis process.

Relevant differences were found while comparing the Korean and white cohorts: in the white cohort, the largest cluster (C1) corresponded to a moderate non-surgical cluster, and the smallest cluster matched the most extreme phenotype (C6), whereas in the Korean external cohort, the largest and smallest clusters corresponded to severe surgical phenotypes (C5 and C3, respectively). We acknowledge that the external Korean cohort is limited (*n* = 186) and that a larger multicentred study would be required to comprehensively assess the Korean population heterogeneity.

Different studies have been conducted to clarify which SCIII subphenotypes can be successfully corrected non-surgically and which require surgical treatment^27^. Kerr et al.^55^ proposed cephalometric yardsticks to support clinical decision making using univariate statistical analysis, concluding that patients exhibiting ANB angles lower than −4°, a maxillomandibular ratio equal to or greater than 0.78, and IMPA angles lower than 83° should be treated surgically. Alternatively, Stellzig-Eisenhauer et al.^48^ performed a stepwise cephalometric variable selection, proposing a four-variable model that aimed to distinguish patients with and without indications for surgical correction. The selected variables were the Wits appraisal, SN, M/M ratio, and lower gonial angle. Our results agree with those of previous studies. The surgical clusters (C5, C3 and C6) found in the present study exhibited ANB angles lower than −4° and maxillomandibular ratios equal to or greater than 0.78. Furthermore, the four variable model points towards some of the most relevant cephalometric variables highlighted in our study. Our results further agree with previous research regarding the morphometric characteristics of the most severe subphenotypes^48,55,56^, representing the average features of severe high-angle SCIII subphenotypes with maxillary retrusion and mandibular prognathism, which were similar to those found in C6.

Notwithstanding, SCIII diagnosis remains a challenging and complex process in clinical practice. Clinical assessment is key for an accurate diagnosis: adult SCIII patients may present facial asymmetries, cross-bites and dental factors such as dual bites and occlusal interferences, which can lead to inaccurate positioning of the mandible^48^. Patients’ expectations toward treatment should also be considered, as well as the risks and limitations of both treatment options. The decision to treat surgically or non-surgically depends on several variables that are not measurable on cephalography and should be carefully assessed in a clinical setting.

Our study advocates for a data-driven approach, with a focus on anatomical landmarks as the central element for cluster identification using GM to avoid biases introduced by cephalometric variables. The adopted methodology further allowed the development of a classification model, based on the white broad adult Iberian population, capable of distinguishing six different subphenotypes with increasing severity: slight (C2), mild (C1 and C4) and severe (C5, C3 and C6). The defined SCIII subphenotypes may help clinicians to develop efficient differential diagnoses through the adoption of a classification diagnostic model (available at: https://tools.istars.pt/sciii/), thereby serving as a copilot for aiding therapeutic decision making (Supplementary Data 1–Fig. H).

## Supplementary information

Transparent Peer Review file

Description of Additional Supplementary Files

Supplementary Data 1

Supplementary Data 2

## Data Availability

The original clinical datasets generated through this study may be available upon reasonable request to the authors (cristina.vft@gmail.com). Access is granted under the following conditions: the associated projects have been approved by the ethical committee, patients’ informed consent is respected, and data use is limited to the approved project and cannot be shared further without additional approval. The requests will be reviewed by the authors, with a response expected within 3 weeks. All processed data generated during the analysis are included in Supplementary Data 2.

## Code availability

All code used for our analysis and to produce figures can be found in our GitHub repository available at https://github.com/istars-fmul/proj-sciii-diagnostic and in the Zenodo archive at 10.5281/zenodo.18755167. This software is licensed under the European Union Public Licence (EUPL) v1.2 or later. For proprietary use, commercial support, or alternative licensing terms, please contact the authors.
