# Supplementary material for: Geometric morphometrics based diagnostic model for Skeletal Class III patients
Source: Commun Med (Lond). 2026 Apr 14;6:340. doi: 10.1038/s43856-026-01557-y (PMC13270103; doi:10.1038/s43856-026-01557-y)
Supplement: Supplementary file 2 — Description of Additional Supplementary Files [file 43856_2026_1557_MOESM2_ESM.pdf]

Description of Additional Supplementary Files

File name: Supplementary Data 1

Description: Additional statistical tables and figures supporting the results presented in the manuscript.

File name: Supplementary Data 2

Description: Processed data generated during the analysis.
