# Supplementary material for: Geometric morphometrics based diagnostic model for Skeletal Class III patients
Source: Commun Med (Lond). 2026 Apr 14;6:340. doi: 10.1038/s43856-026-01557-y (PMC13270103; doi:10.1038/s43856-026-01557-y)
Supplement: Supplementary file 3 — Supplementary Data 1 [file 43856_2026_1557_MOESM3_ESM.docx]

**Supplementary Data 1**

**Geometric morphometrics based diagnostic model for Skeletal Class III patients.**

Maria Cristina Faria-Teixeira^1, 2, #^, Inês M. N. Carvalho^2, #^, Alexandra Dehesa-Santos^1^, Francisco Salvado e Silva^2^, Helena Afonso Agostinho^3^, Francisco Do Vale^4^, António Vaz-Carneiro^2, 5^, Leixuri De Frutos-Valle^1^, Shin-Jae Lee^6^, Joao C. Guimaraes^2,^*, and Alejandro Iglesias-Linares^1,7,^*

1. Radiological landmarks and cephalometric measurements

Table A.1 – Landmark coordinates description.

| Landmark Coordinates | |
| --- | --- |
| Cranial Base | Nasion [N] |
|  | Sella [S] |
|  | Basion [Ba] |
| Maxilla | Posterior Nasal Spine [PNS] |
|  | Downs A Point [A] |
| Mandible | Downs B Point [B] |
|  | Pogonion [Pg] |
|  | Menton [Me] |
|  | Gonion [Go] |
|  | Ramus point [Ramus] |
|  | Distal aspect of condyle [21] |
|  | Condylion [Co] |

Table A.2 – Cephalometric variables description.

| Cephalometric Variables | | |
| --- | --- | --- |
| Angular | FH-SN (º) | Angle formed by Frankfort plane (FH) and Sella (S)- Nasion (N) plane |
|  | SNA (º) | Angle formed by Sella (S), Nasion (N) and point A Downs (A) |
|  | SNB (º) | Angle formed by Sella (S), Nasion (N) and point B Downs (B) |
|  | ANB (º) | Angle formed by Nasion (N), point A Downs (A) and point B Downs (B) |
|  | SND (º) | Angle formed by Sella (S), Nasion (N) and point D |
|  | Y-Axis (SGn-SN) (º) | Angle formed by Sella (S)- Gnathion (Gn) plane and Sella (S)- Nasion (N) plane |
|  | SN-GoGn (º) | Angle formed by Sella (S)- Nasion (N) plane and Gonion (Go)-Gnathion (Gn) plane |
|  | Cranio-Mx Base/SN-Palatal Plane (º) | Angle formed by Sella (S)- Nasion (N) plane and palatal plane |
|  | Occ Plane to SN (º) | Angle formed by the occlusal plane and Sella (S)- Nasion (N) plane |
|  | Occ Plane to FH (º) | Angle formed by the occlusal plane and Frankfort plane (FH) |
|  | Facial Axis-Ricketts (NaBa-PtGn) (º) | Angle formed by Nasion (N)-Basion (Ba) plane and Pterigoid (Pt)-Gnation (Gn) plane |
|  | FMA (MP-FH) (º) | Angle of mandibular plane (FMA). Angle formed by mandibular plane (MP) and Frankfort plane (FH) |
|  | Lower Face Height (ANS-Xi-Pm) (º) | Angle formed by the anterior nasal spine (ANS), Xi point and suprapogonion (Pm) |
|  | Facial Angle (FH-NPo) (º) | Angle formed by Frankfort plane (FH) and Nasion (N)- Pogonion (Pg) plane |
|  | N-A-Pg (º) | Angle formed by Nasion (N), point A Downs (A) and Pogonion (Pg) |
|  | Facial Taper (º) | Angle formed by the mandibular plane and facial plane |
|  | Gonial/Jaw Angle (Ar-Go-Me) (º) | Angle formed by Articulare (Ar), Gonion (Go) and Menton (Me) |
|  | Upper Gonial Angle (Ar-Go-Na) (º) | Angle formed by Articulare (Ar), Gonion (Go) and Nasion (Na) |
|  | Lower Gonial Angle (Na-Go-Me) (º) | Angle formed by Nasion (NA), Gonion (Go) and Menton (Me) |
|  | Articular Angle (º) | Angle formed by Sella (S), Articulare (Ar) and Gonion (Go) |
|  | Saddle/Sella Angle (SN-Ar) (º) | Angle formed by Sella (S), Nasion (Na) and Articulare (Ar).  Cranial base angle |
|  | Superior Angle SN-AB (º) | Angle formed by Sella (S)- Nasion (N) plane and point A Downs (A)- point B Downs (B) plane |
|  | Rp-FH (º) | Angle formed by ramus plane (Rp) and Frankfort plane (FH) |
| Linear | Anterior Cranial Base (SN) (mm) | Distance between Sella (S) and Nasion (Na) |
|  | Anterior Face Height (NaMe) (mm) | Distance between Nasion (Na) and Menton (Me) |
|  | Upper Face Height (N-ANS) (mm) | Distance between Nasion (Na) and Anterior Nasal Spine (ANS) |
|  | Lower Face Height (ANS-Me) (mm) | Distance between Anterior Nasal Spine (ANS) and Menton (Me) |
|  | Posterior Cranial Base (S-Ar) (mm) | Distance between Sella (S) and Articulare (Ar) |
|  | Posterior Face Height (SGo) (mm) | Distance between Sella (S) and Gonion (Go) |
|  | Ramus Height (Ar-Go) (mm) | Distance between Articulare (Ar) and Gonion (Go) |
|  | Co-Go (mm) | Distance between Condylion (Co) and Gonion (Go) |
|  | Convexity (A-NPo) (mm) | Distance from point A Downs (A) to Nasion (N)- Pogonion (Pg) line |
|  | Maxillary Skeletal (A-Na Perp) (mm) | Distance from point A Downs (A) to the line perpendicular to the Frankfort plane (FH) via Nasion (N perp) |
|  | Midface Length (Co-A) (mm) | Distance between Condylion (Co) and point A Downs (A) |
|  | Ar-A (mm) | Distance between Articulare (Ar) and point A Downs (A) |
|  | Maxillary length (ANS-PNS) (mm) | Distance between the Anterior Nasal Spine (ANS) and the Posterior Nasal Spine (PNS) |
|  | Pog-NB (mm) | Distance between Pogonion (Pg) to Nasion (Na) - point B Downs (B) line |
|  | Mand. Skeletal (Pg-Na Perp) (mm) | Distance between point B Downs (B) to the line perpendicular to the Frankfort plane (FH) via Nasion (N perp) |
|  | Mandibular Body Length (Go-Gn) (mm) | Distance between Gonion (Go) and Gnathion (Gn) |
|  | Length of Mand Base (Go-Pg) (mm) | Distance between Gonion (Go) and Pogonion (Pg) |
|  | Mandibular length (Co-Gn) (mm) | Distance between Condylion (Co) and Gnathion (Gn) |
|  | Co-B1 Total mand (mm) | Distance between Condylion (Co) and point B Downs (B) |
|  | Ar - Gn (mm) | Distance between Articulare (Ar) and Gnathion (Gn) |
|  | Basal Width (mm) | Horizontal distance of the symphysis at poin B Downs (B) level |
|  | Mx/Md diff (Co-Gn-Co-A) (mm) | Difference between the Condylion (Co) and Gnathion (Gn) distance and the Condylion (Co) and point A Downs (A) Co-Gn-Co-A (mm) (Mx/Md diff) (A) distance |
|  | Wits Appraisal (mm) | distance between point A Downs (A) and point B Downs (B) projected in the occlusal plane |
| Proportional | P-A Face Height(S-Go/N-Me) (%) | Proportion exiting between Sella (S)- Gonion (Go) distance and the Nasion (N) and Menton (Me) distance |
|  | PFH:AFH (%) | Ratio of Posterior Facial weight (PFH) to Anterior Facial Height (AFH) |
|  | S-Ar/Ar-Go (%) | Ratio of Posterior Cranial Base (S-Ar) to ramus height (Ar-Go) |
|  | UFH (N-ANS/(N-ANS+ANS-Me)) (%) | Proportion of Upper Facial Height |
|  | LFH/TFH (ANS-Me:N-Me) (%) | Ratio of Lower Facial Height (LFH) to Total Facial Height (TFH) |
|  | Face Ht Ratio (N-ANS/ANS-Me) (%) | Total Facial Height Ratio |
|  | SN/GoMe (%) | Proportion between Sella (S)- Nasion (N) line and Gonion (Go)-Menton (me) line |

1. *SCIII Southern European Sample Characteristics*

*Table B.1 – Summary of the study SCIII Southern European sample characteristics.*

| Count | Iberian Origin | | Gender | | Age  (years) |
| --- | --- | --- | --- | --- | --- |
|  | Portuguese | Spanish | Women | Men |  |
| 655 | 266 | 389 | 315 | 340 | 30.08±10.45 |

Table B.2 – Mean and standard deviations of cohort's Cephalometric Variables.

| Cephalometric_Variables | mean_sd | Cephalometric_Variables | mean_sd |
| --- | --- | --- | --- |
| FH - SN (º) | 10.39 ± 3.55 | Posterior Cranial Base (S-Ar) (mm) | 34.2 ± 7.98 |
| SNA (º) | 79.49 ± 3.97 | Posterior Face Height (SGo) (mm) | 82.43 ± 19.33 |
| SNB (º) | 83.02 ± 4.66 | Ramus Height (Ar-Go) (mm) | 52.89 ± 13.53 |
| ANB (º) | -3.53 ± 3.58 | Co-Go (mm) | 68.53 ± 18.32 |
| SND (º) | 80.45 ± 4.44 | Convexity (A-NPo) (mm) | -4.3 ± 3.95 |
| Y-Axis (SGn-SN) (º) | 66.56 ± 4.63 | Maxillary Skeletal (A-Na Perp) (mm) | -0.08 ± 4.59 |
| SN - GoGn (º) | 31.6 ± 7.02 | Midface Length (Co-A) (mm) | 87.22 ± 19.54 |
| Cranio-Mx Base/SN-Palatal Plane (º) | 8.12 ± 3.84 | Ar - A (mm) | 85.63 ± 18.67 |
| Occ Plane to SN (º) | 15.16 ± 5.23 | Maxillary length (ANS-PNS) (mm) | 55.13 ± 13.39 |
| Occ Plane to FH (º) | 4.78 ± 5.03 | Pog - NB (mm) | 0.96 ± 3.14 |
| Facial Axis-Ricketts (NaBa-PtGn)(º) | 91.36 ± 5.36 | Mand. Skeletal (Pg-Na Perp) (mm) | 8.39 ± 10.7 |
| FMA (MP-FH) (º) | 23.91 ± 6.83 | Mandibular Body Length (Go-Gn)(mm) | 88.34 ± 20.94 |
| Lower Face Height (ANS-Xi-Pm)(º) | 44.62 ± 5.96 | Length of Mand Base (Go-Pg)(mm) | 77.11 ± 17.64 |
| Facial Angle (FH-NPo) (º) | 93.91 ± 4.3 | Mandibular length (Co-Gn)(mm) | 129.29 ± 30.94 |
| N-A-Pg (º) | -8.15 ± 6.88 | Co-B1 Total mand (mm) | 126.72 ± 30.54 |
| Facial Taper (º) | 62.17 ± 5.89 | Ar - Gn (mm) | 121.93 ± 28.68 |
| Gonial/Jaw Angle (Ar-Go-Me) (º) | 127.7 ± 8.47 | Basal Width (mm) | 7.34 ± 2.44 |
| Upper Gonial Angle (Ar-Go-Na) (º) | 50.7 ± 4.59 | Mx/Md diff (Co-Gn - Co-A) (mm) | 42.07 ± 13.4 |
| Lower Gonial Angle (Na-Go-Me) (º) | 77 ± 6.71 | Wits Appraisal (mm) | -9.81 ± 6.85 |
| Articular Angle (º) | 142.06 ± 7.55 | P-A Face Height(S-Go/N-Me) (%) | 64.85 ± 5.68 |
| Saddle/Sella Angle (SN-Ar) (º) | 124.54 ± 5.71 | PFH:AFH (%) | 53.73 ± 5.56 |
| Superior Angle SN-AB (º) | 88.22 ± 9.29 | S-Ar/Ar-Go (%) | 65.66 ± 10.34 |
| Rp-FH (º) | 76.21 ± 6.05 | UFH (N-ANS/(N-ANS+ANS-Me)) (%) | 42.93 ± 2.55 |
| Anterior Cranial Base (SN) (mm) | 71.17 ± 15.49 | LFH/TFH (ANS-Me:N-Me) (%) | 57.14 ± 2.59 |
| Anterior Face Height (NaMe) (mm) | 127.41 ± 29.13 | Face Ht Ratio (N-ANS/ANS-Me) (%) | 0.75 ± 0.09 |
| Upper Face Height (N-ANS) (mm) | 54.86 ± 12.01 | SN/GoMe (%) | 97.59 ± 9.06 |
| Lower Face Height (ANS-Me) (mm) | 73.3 ± 18.15 | ANS-PNS/Me-Go (%) | 0.75 ± 0.09 |

*Table B.3 – Results of two-sided ANOVA assessing the effect of Landmark, Operator, and their interaction on measurement variability.*

| **Source** | **Df** | **Sum Sq** | **Mean Sq** | **F value** | **Pr(>F)** |
| --- | --- | --- | --- | --- | --- |
| Landmark | 23 | 1328359 | 57755 | 2466.240 | <2e-16 |
| Operator | 1 | 0 | 0 | 0.000 | 1.000 |
| Landmark:Operator | 23 | 282 | 12 | 0.523 | 0.968 |
| Residuals | 432 | 10117 | 23 |  |  |

Table B.4 – Landmarks “Single_fixed_raters” Intraclass Correlation (ICC3) reliability results. F-tests are two-sided.

|  | **ICC** | **F** | **df1** | **df2** | **pvalue** | **lower bound** | **upper bound** |
| --- | --- | --- | --- | --- | --- | --- | --- |
| **X_Sella** | 0.92 | 22.81 | 9 | 9 | 0 | 0.7 | 0.98 |
| **Y_Sella** | 0.81 | 9.78 | 9 | 9 | 0 | 0.42 | 0.95 |
| **X_Basion** | 0.74 | 6.59 | 9 | 9 | 0 | 0.24 | 0.93 |
| **Y_Basion** | 0.81 | 9.51 | 9 | 9 | 0 | 0.41 | 0.95 |
| **X_PNS** | 0.35 | 2.1 | 9 | 9 | 0.14 | -0.32 | 0.79 |
| **Y_PNS** | 0.96 | 44.49 | 9 | 9 | 0 | 0.83 | 0.99 |
| **X_A Point** | 0.72 | 6.22 | 9 | 9 | 0.01 | 0.21 | 0.92 |
| **Y_A Point** | 0.88 | 15.27 | 9 | 9 | 0 | 0.58 | 0.97 |
| **X_B Point** | 0.77 | 7.59 | 9 | 9 | 0 | 0.31 | 0.94 |
| **Y_B Point** | 0.8 | 8.94 | 9 | 9 | 0 | 0.38 | 0.95 |
| **X_Pogonion** | 0.85 | 12.18 | 9 | 9 | 0 | 0.5 | 0.96 |
| **Y_Pogonion** | 0.91 | 21.61 | 9 | 9 | 0 | 0.69 | 0.98 |
| **X_Menton** | 0.88 | 16.38 | 9 | 9 | 0 | 0.61 | 0.97 |
| **Y_Menton** | 0.98 | 101.6 | 9 | 9 | 0 | 0.92 | 1 |
| **X_Gonion** | 0.8 | 9.25 | 9 | 9 | 0 | 0.39 | 0.95 |
| **Y_Gonion** | 0.76 | 7.26 | 9 | 9 | 0 | 0.29 | 0.93 |
| **X_Ramus Point** | 0.72 | 6.14 | 9 | 9 | 0.01 | 0.21 | 0.92 |
| **Y_Ramus Point** | 0.63 | 4.41 | 9 | 9 | 0.02 | 0.05 | 0.89 |
| **X_Distal Aspect of Condyle** | 0.83 | 10.78 | 9 | 9 | 0 | 0.46 | 0.95 |
| **Y_Distal Aspect of Condyle** | 0.68 | 5.3 | 9 | 9 | 0.01 | 0.14 | 0.91 |
| **X_Condylion** | 0.08 | 1.18 | 9 | 9 | 0.4 | -0.55 | 0.65 |
| **Y_Condylion** | 0.51 | 3.11 | 9 | 9 | 0.05 | -0.13 | 0.85 |
| **X_Nasion** | 0.98 | 122.0 | 9 | 9 | 0 | 0.94 | 1 |
| **Y_Nasion** | 0.94 | 34.91 | 9 | 9 | 0.00 | 0.79 | 0.99 |

1. *Generalized Procrustes Analysis*

Table C.1 – Summary of the 12 Landmark Coordinates (mean ± sd) after the GPA.

| Landmark | X | Y |
| --- | --- | --- |
| A Point | 42.98 ± 3.83 | 3.68 ± 3.13 |
| B Point | 48.63 ± 2.36 | -38.82 ± 3.29 |
| Basion | -52.78 ± 4.10 | 13.24 ± 3.42 |
| Condylion | -40.41 ± 2.64 | 35.01 ± 2.79 |
| Distal Aspect of Condyle | -44.91 ± 2.09 | 29.54 ± 3.32 |
| Gonion | -24.43 ± 4.68 | -32.74 ± 4.64 |
| Menton | 43.64 ± 2.78 | -64.15 ± 2.76 |
| Nasion | 44.02 ± 3.81 | 66.08 ± 4.62 |
| PNS | -6.33 ± 3.07 | 8.93 ± 2.32 |
| Pogonion | 50.55 ± 2.33 | -56.75 ± 3.23 |
| Ramus Point | -33.22 ± 4.12 | -18.09 ± 4.76 |
| Sella | -27.74 ± 2.34 | 54.07 ± 2.43 |

Table C.2 – Summary of the Procrustes Residuals (mean ± sd)

| Landmark | X | Y |
| --- | --- | --- |
| A Point | 0.00 ± 3.83 | 0.00 ± 3.13 |
| B Point | 0.00 ± 2.36 | 0.00 ± 3.29 |
| Basion | 0.00 ± 4.10 | 0.00 ± 3.42 |
| Condylion | 0.00 ± 2.64 | 0.00 ± 2.79 |
| Distal Aspect of Condyle | 0.00 ± 2.09 | 0.00 ± 3.32 |
| Gonion | 0.00 ± 4.68 | 0.00 ± 4.64 |
| Menton | 0.00 ± 2.78 | 0.00 ± 2.76 |
| Nasion | 0.00 ± 3.81 | 0.00 ± 4.62 |
| PNS | 0.00 ± 3.07 | 0.00 ± 2.32 |
| Pogonion | 0.00 ± 2.33 | 0.00 ± 3.23 |
| Ramus Point | 0.00 ± 4.12 | 0.00 ± 4.76 |
| Sella | 0.00 ± 2.34 | 0.00 ± 2.43 |

1. *Unsupervised learning-guided identification of SCIII subphenotypes*


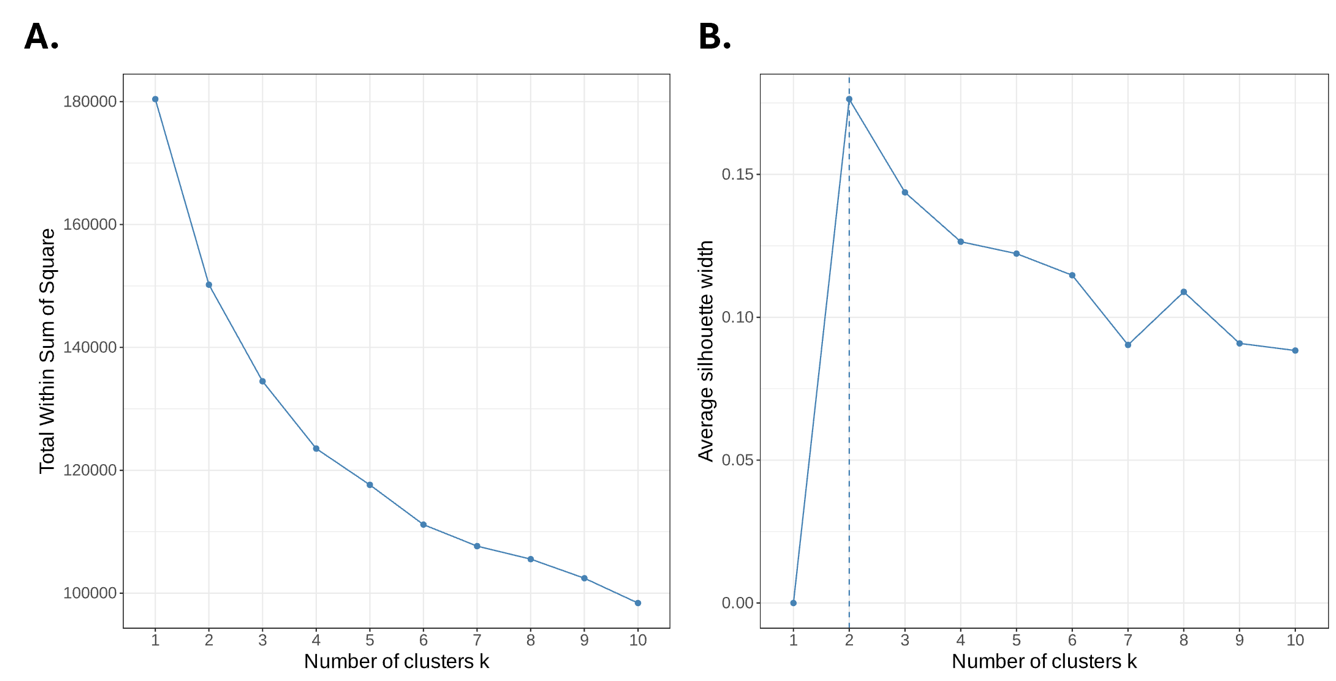


Figure D.1 – Clustering evaluation metrics for different numbers of clusters. (A) Within-cluster sum of squares and (B) Average Silhouette width as a function of the number of clusters (k).


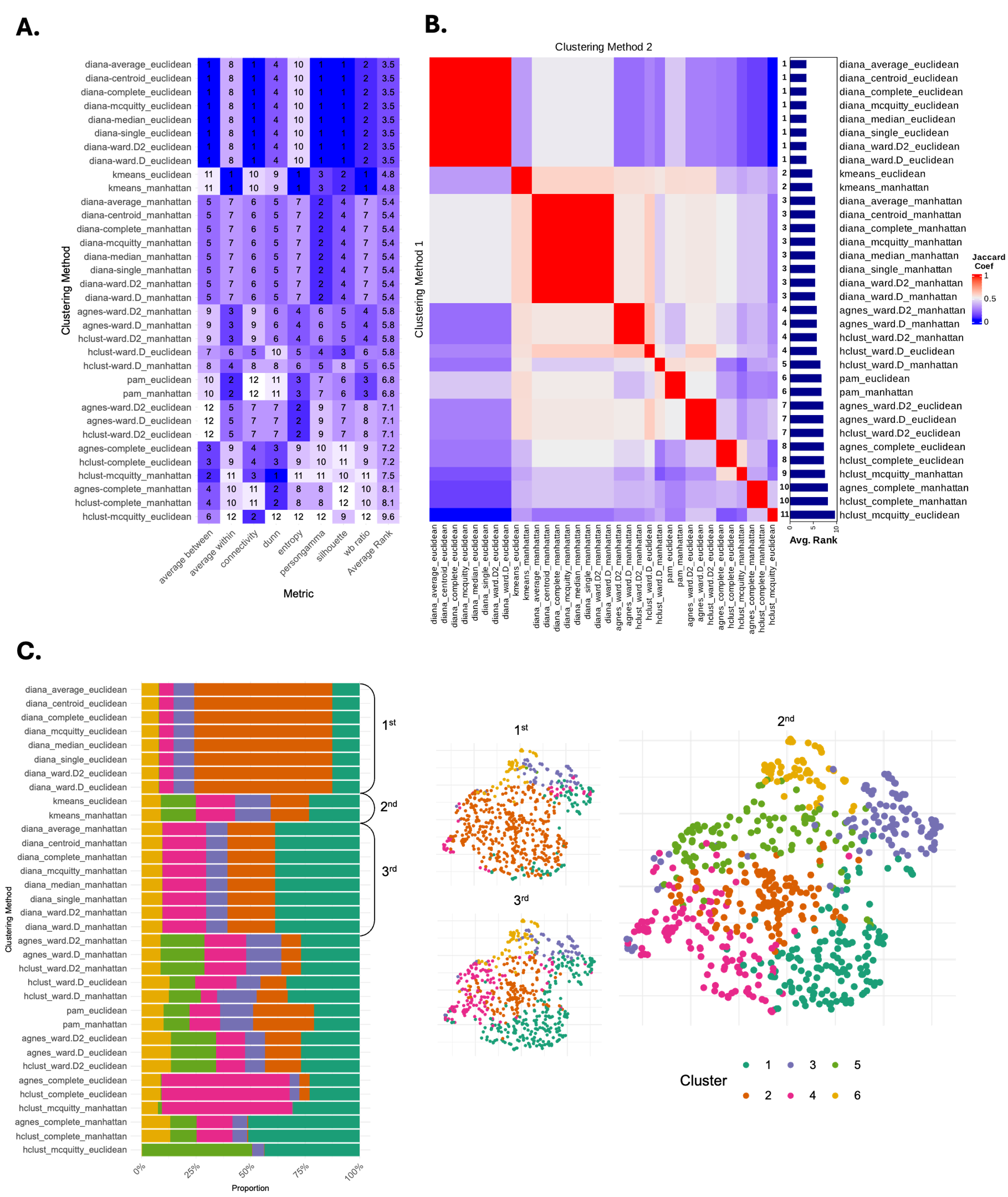


Figure D.2 – Detailed analysis of algorithm selection for solution with 6 clusters. The results show that K-means achieves the best average ranking across various clustering metrics while maintaining a balanced assignment of patients to the different clusters. (A) Ranking of clustering algorithms based on different evaluation metrics. (B) Comparison of clustering results overlap using different algorithms, as evaluated by the maximum Jaccard coefficient. (C) The distribution of patients per cluster demonstrates that K-means effectively partitions the data into six meaningful clusters (left), avoiding sparsely populated clusters (i.e., clusters with fewer than 5 patients). UMAP representation of the different patients and respective clustering assignment (represented by the different colors) for the top 3 ranked clustering algorithms (right).


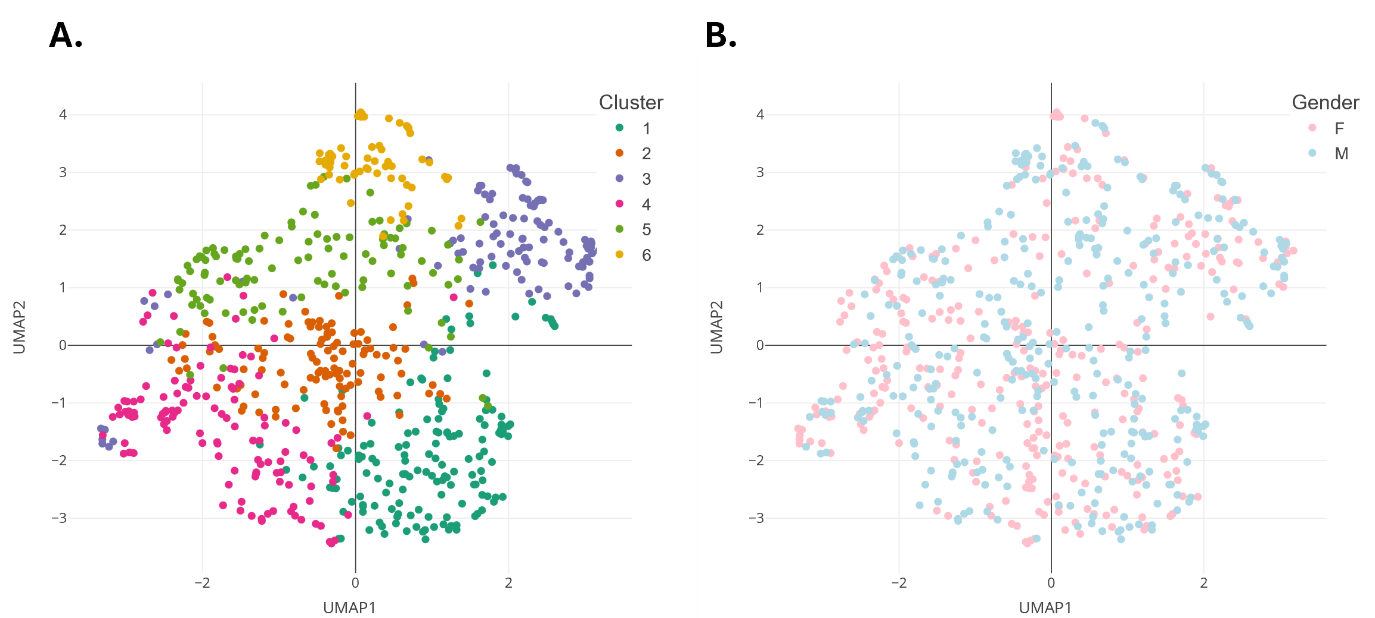


Figure D.3 – UMAP representation of the distribution of different patients per cluster and gender. (A) UMAP representation of the patients’ Procrustes coordinates residuals and the cluster assignment (represented by the different colors), each dot representing a patient; (B) UMAP representation of the patients’ Procrustes coordinates residuals and the gender (M, male, in cyan; F, female, in red), each dot representing a patient.


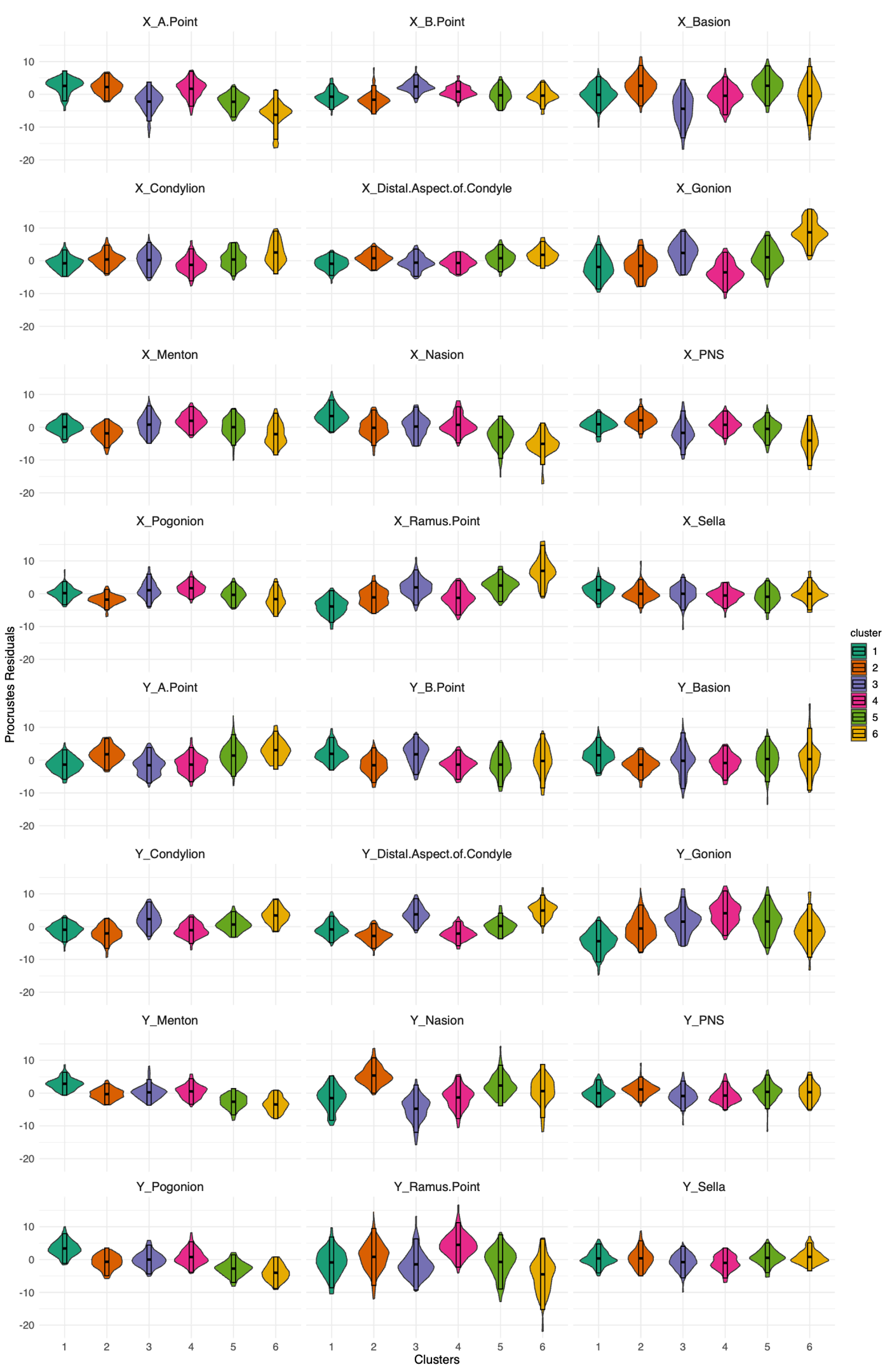
 *Figure D.4 – Violin plot of the Procrustes’ residuals for the different landmarks per cluster.*


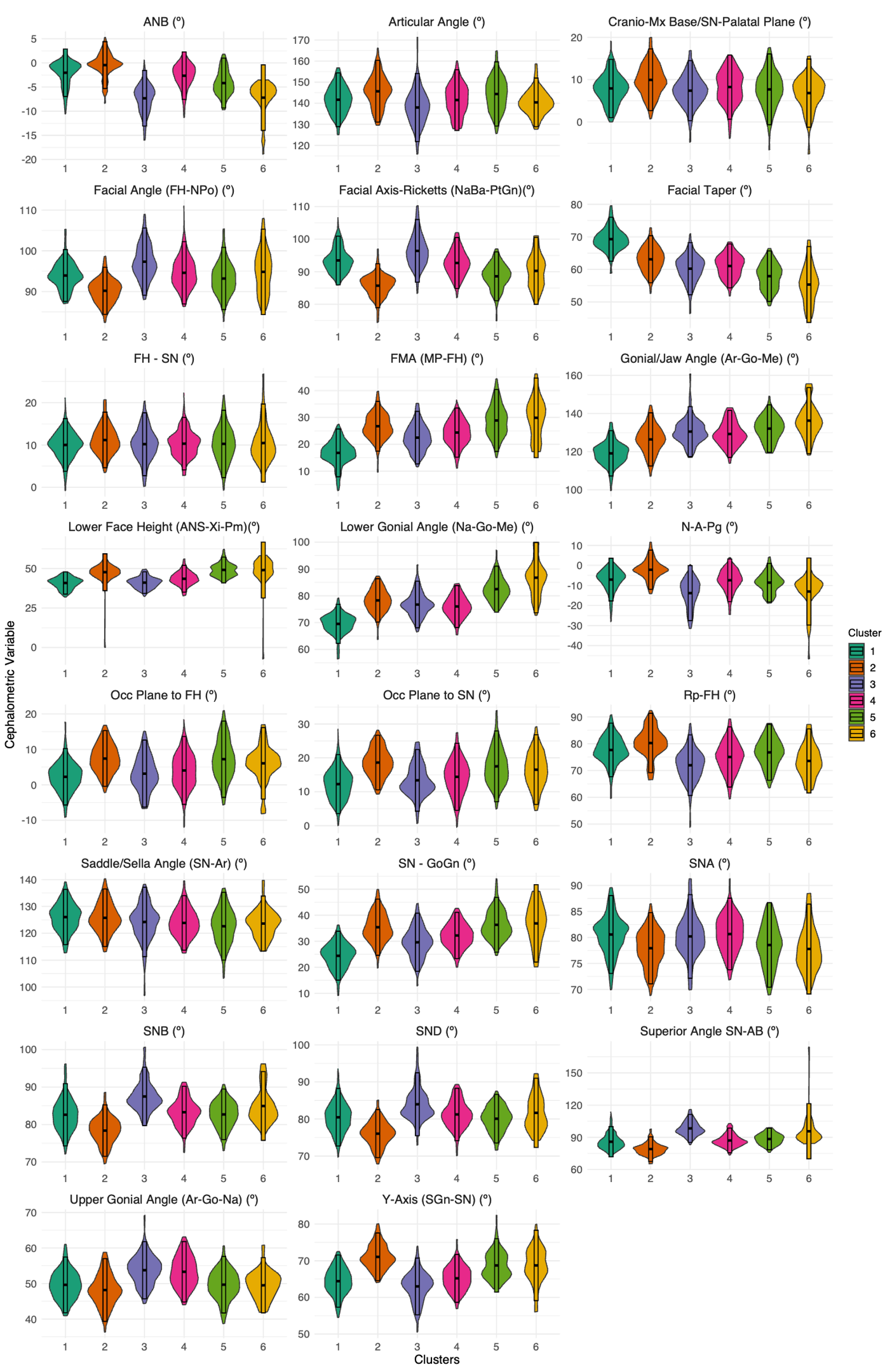
 *Figure D.5.A – Violin plot of the angular cephalometric variables per cluster.*


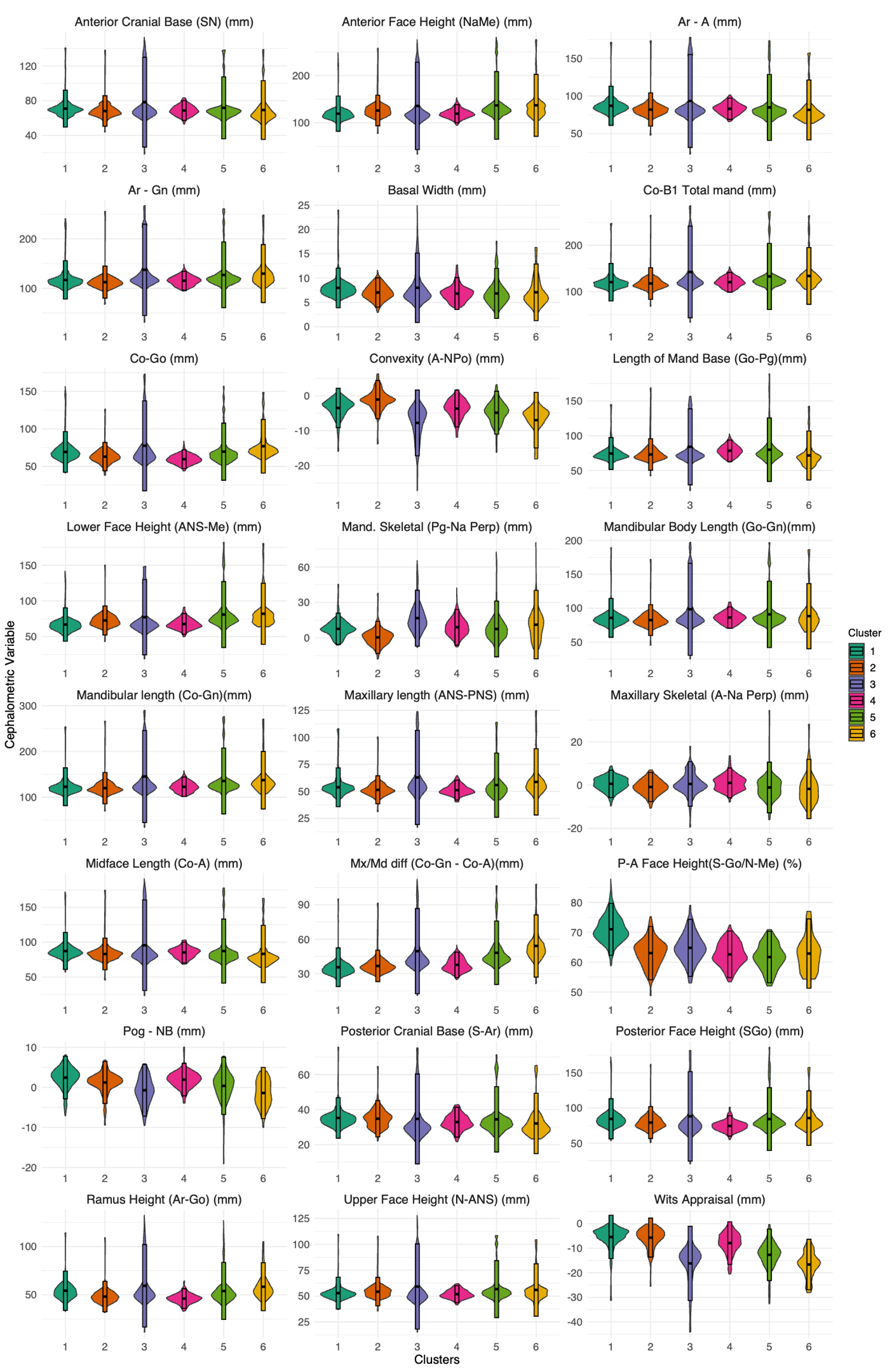
 *Figure D.5.B – Violin plot of the linear cephalometric variables per cluster.*


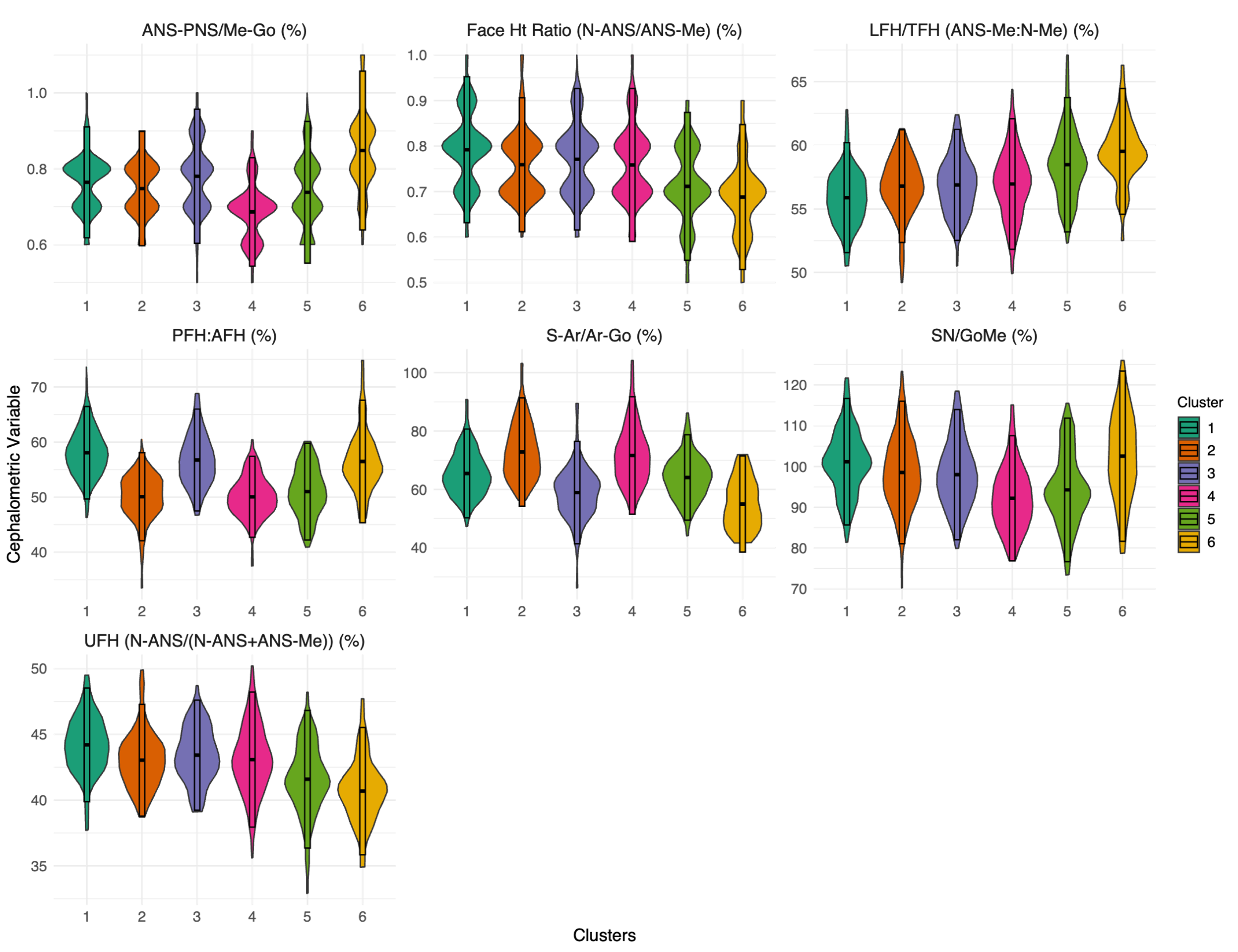


Figure D.5.C – Violin plot of the proportional cephalometric variables per cluster.


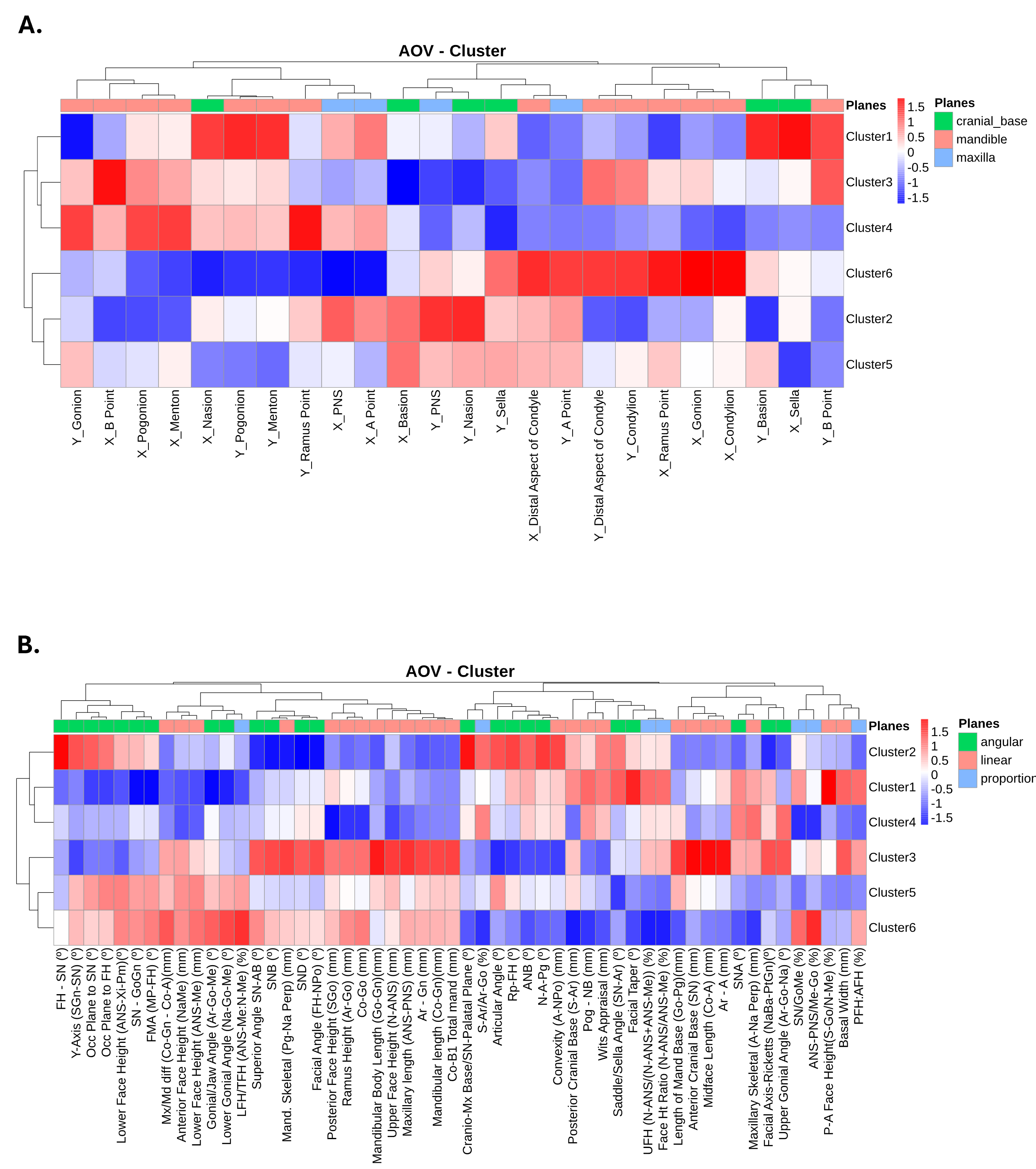


*Figure D.6 – Visualization of shifts in landmark coordinates and cephalometric variables across clusters using ANOVA scaled coefficients (A) Heatmap of the ANOVA scaled coefficients, indicating per cluster which landmark coordinates are positively or negatively shifted, in red and blue, respectively, in relation to the mean of the cohort; (B) Heatmap of the ANOVA scaled coefficients, indicating per cluster which cephalometric variables are increased or decreased, in red and blue, respectively, in relation to the mean of the cohort.*


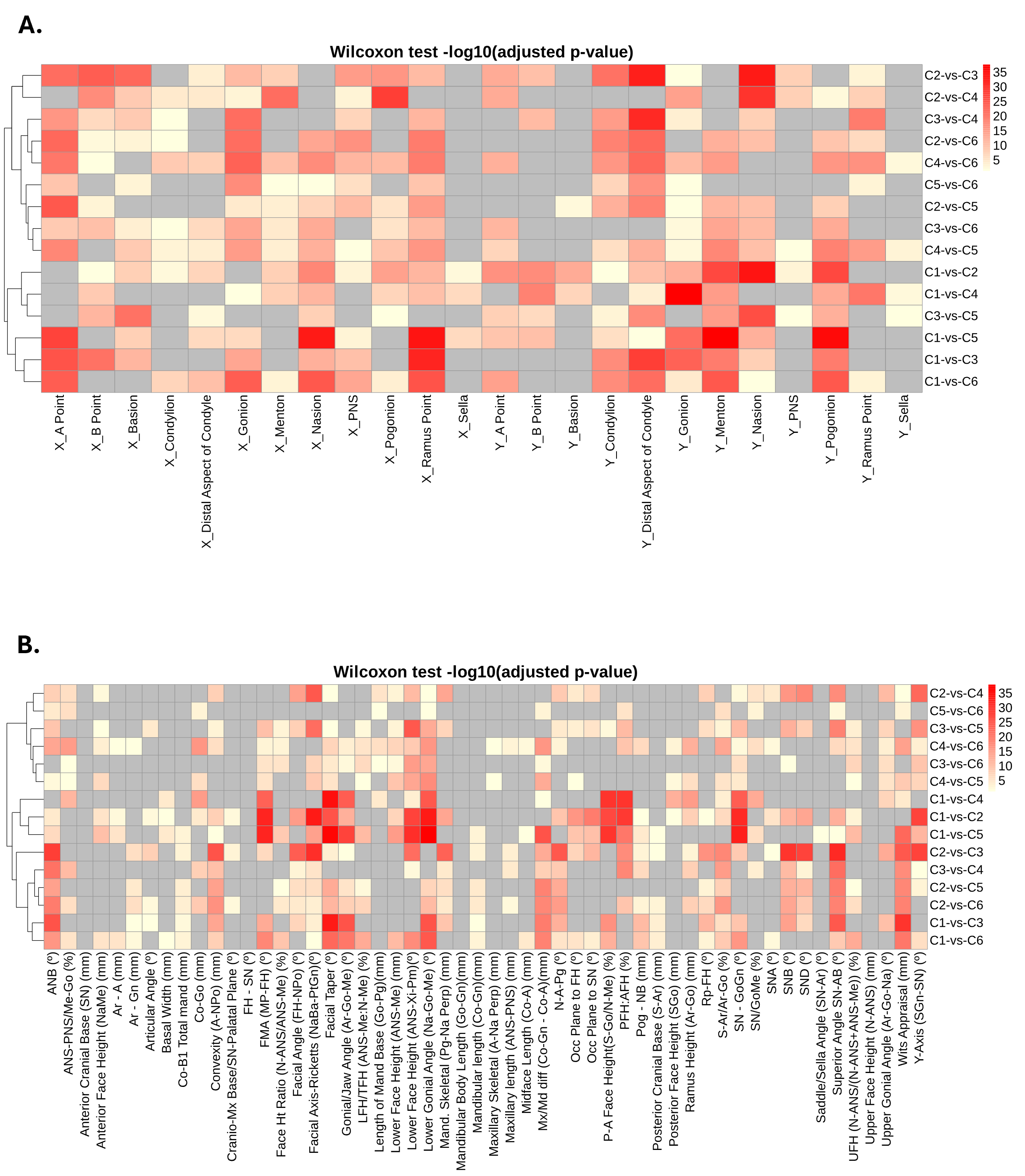
 *Figure D.7 – Pairwise cluster comparisons for landmarks and cephalometric variables. (A) Heatmap of pairwise cluster Wilcoxon -log10(adjusted p-value) for the landmark coordinates; (B) Heatmap of pairwise cluster Wilcoxon -log10(adjusted p-value) for the cephalometric measurements. Gray indicates adjusted p-values greater than 0.05, showing no significant difference between cluster pairs for that variable. A yellow-to-red scale represents p-values below the significance threshold (0.05), with red indicating lower adjusted p-values than yellow.*

Table D.1 – Rotated Landmark Coordinates per cluster (mean ± sd)

|  | Cluster 1 | Cluster 2 | Cluster 3 | Cluster 4 | Cluster 5 | Cluster 6 |
| --- | --- | --- | --- | --- | --- | --- |
| X_Sella | -26.65 ± 2.09 | -27.74 ± 2.16 | -27.74 ± 2.48 | -28.29 ± 1.97 | -28.68 ± 2.44 | -27.75 ± 2.46 |
| Y_Sella | 54.43 ± 2.21 | 54.44 ± 2.67 | 53.31 ± 2.41 | 53.03 ± 2.29 | 54.62 ± 2.25 | 54.9 ± 2.12 |
| X_Basion | -52.91 ± 2.76 | -50.16 ± 3.07 | -57.17 ± 4.43 | -53.22 ± 2.89 | -50.18 ± 3.05 | -53.29 ± 4.48 |
| Y_Basion | 14.73 ± 2.72 | 11.84 ± 2.33 | 13.08 ± 4.25 | 12.38 ± 2.63 | 13.6 ± 3.46 | 13.52 ± 4.71 |
| X_PNS | -5.43 ± 1.89 | -4.22 ± 2.08 | -8.04 ± 3.33 | -5.56 ± 2.09 | -6.84 ± 2.48 | -10.38 ± 3.8 |
| Y_PNS | 8.9 ± 2.03 | 10.02 ± 1.91 | 8.04 ± 2.28 | 8.2 ± 2.17 | 9.31 ± 2.56 | 9.22 ± 2.62 |
| X_A.Point | 45.54 ± 2.29 | 45.19 ± 2.1 | 40.76 ± 2.96 | 44.66 ± 2.65 | 40.68 ± 2.31 | 36.75 ± 3.74 |
| Y_A.Point | 2.33 ± 2.25 | 5.47 ± 2.42 | 2.13 ± 2.71 | 2.33 ± 2.6 | 5.1 ± 3.18 | 6.72 ± 2.89 |
| X_B.Point | 47.92 ± 1.95 | 46.97 ± 2.18 | 50.99 ± 1.8 | 49.45 ± 1.6 | 48.35 ± 2.34 | 48.24 ± 2.06 |
| Y_B.Point | -36.85 ± 2.49 | -40.35 ± 2.65 | -37.03 ± 3.08 | -40.18 ± 2.23 | -40.14 ± 3.38 | -39.03 ± 4.13 |
| X_Pogonion | 50.73 ± 1.77 | 48.74 ± 1.55 | 51.59 ± 2.45 | 52.26 ± 1.71 | 50.2 ± 1.98 | 48.88 ± 2.63 |
| Y_Pogonion | -53.37 ± 2.27 | -57.44 ± 2.12 | -56.75 ± 2.2 | -55.98 ± 2.33 | -59.52 ± 2.12 | -60.76 ± 2.41 |
| X_Menton | 43.73 ± 1.89 | 41.78 ± 2.2 | 44.45 ± 2.85 | 45.6 ± 2.17 | 43.69 ± 2.76 | 41.55 ± 3.17 |
| Y_Menton | -61.32 ± 1.75 | -64.47 ± 1.6 | -63.92 ± 1.96 | -63.65 ± 1.94 | -66.79 ± 2.01 | -67.62 ± 2.18 |
| X_Gonion | -26.29 ± 3.37 | -25.95 ± 3.13 | -22.06 ± 3.33 | -27.94 ± 3.04 | -23.35 ± 3.33 | -15.74 ± 3.55 |
| Y_Gonion | -37.19 ± 3.16 | -33.31 ± 3.59 | -31.22 ± 3.75 | -28.63 ± 3.4 | -31.17 ± 4.03 | -33.93 ± 4.05 |
| X_Ramus.Point | -37.12 ± 2.41 | -34.33 ± 2.46 | -31.31 ± 2.68 | -34.48 ± 2.62 | -30.73 ± 2.45 | -26.25 ± 3.88 |
| Y_Ramus.Point | -18.95 ± 3.87 | -17.28 ± 4.34 | -19.56 ± 3.9 | -13.61 ± 3.39 | -18.81 ± 4.13 | -22.58 ± 5.37 |
| X_Distal.Aspect.of.Condyle | -45.8 ± 1.79 | -44.14 ± 1.79 | -45.5 ± 2.08 | -45.56 ± 1.74 | -44.12 ± 2.07 | -43.12 ± 2.04 |
| Y_Distal.Aspect.of.Condyle | 28.68 ± 2.01 | 26.69 ± 1.89 | 33.28 ± 2.4 | 27.42 ± 1.85 | 29.75 ± 1.95 | 34.44 ± 2.34 |
| X_Condylion | -41.17 ± 2.02 | -40 ± 2.16 | -40.2 ± 2.67 | -41.66 ± 2.44 | -39.99 ± 2.53 | -37.88 ± 3.26 |
| Y_Condylion | 34.05 ± 1.86 | 32.94 ± 2.27 | 37.31 ± 2.62 | 33.92 ± 2.02 | 35.68 ± 1.96 | 38.4 ± 2.42 |
| X_Nasion | 47.45 ± 2.43 | 43.86 ± 2.7 | 44.22 ± 2.98 | 44.76 ± 2.76 | 40.98 ± 3.22 | 38.98 ± 3.17 |
| Y_Nasion | 64.56 ± 3.39 | 71.45 ± 2.69 | 61.33 ± 3.6 | 64.76 ± 3.22 | 68.37 ± 3.1 | 66.72 ± 4.06 |

Table D.2 – Procrustes Residuals per cluster (mean ± sd)

|  | Cluster 1 | Cluster 2 | Cluster 3 | Cluster 4 | Cluster 5 | Cluster 6 |
| --- | --- | --- | --- | --- | --- | --- |
| X_Sella | 1.08 ± 2.09 | 0 ± 2.16 | 0 ± 2.48 | -0.55 ± 1.97 | -0.94 ± 2.44 | -0.01 ± 2.46 |
| X_Basion | -0.14 ± 2.76 | 2.62 ± 3.07 | -4.39 ± 4.43 | -0.45 ± 2.89 | 2.59 ± 3.05 | -0.51 ± 4.48 |
| X_PNS | 0.9 ± 1.89 | 2.12 ± 2.08 | -1.71 ± 3.33 | 0.77 ± 2.09 | -0.51 ± 2.48 | -4.05 ± 3.8 |
| X_A.Point | 2.56 ± 2.29 | 2.21 ± 2.1 | -2.22 ± 2.96 | 1.68 ± 2.65 | -2.3 ± 2.31 | -6.23 ± 3.74 |
| X_B.Point | -0.71 ± 1.95 | -1.65 ± 2.18 | 2.36 ± 1.8 | 0.82 ± 1.6 | -0.27 ± 2.34 | -0.39 ± 2.06 |
| X_Pogonion | 0.18 ± 1.77 | -1.81 ± 1.55 | 1.04 ± 2.45 | 1.71 ± 1.71 | -0.35 ± 1.98 | -1.67 ± 2.63 |
| X_Menton | 0.09 ± 1.89 | -1.87 ± 2.2 | 0.81 ± 2.85 | 1.96 ± 2.17 | 0.05 ± 2.76 | -2.09 ± 3.17 |
| X_Gonion | -1.86 ± 3.37 | -1.52 ± 3.13 | 2.37 ± 3.33 | -3.51 ± 3.04 | 1.08 ± 3.33 | 8.69 ± 3.55 |
| X_Ramus.Point | -3.9 ± 2.41 | -1.11 ± 2.46 | 1.91 ± 2.68 | -1.26 ± 2.62 | 2.49 ± 2.45 | 6.97 ± 3.88 |
| X_Distal.Aspect.of.Condyle | -0.89 ± 1.79 | 0.77 ± 1.79 | -0.59 ± 2.08 | -0.65 ± 1.74 | 0.79 ± 2.07 | 1.79 ± 2.04 |
| X_Condylion | -0.75 ± 2.02 | 0.42 ± 2.16 | 0.21 ± 2.67 | -1.25 ± 2.44 | 0.42 ± 2.53 | 2.54 ± 3.26 |
| X_Nasion | 3.43 ± 2.43 | -0.16 ± 2.7 | 0.2 ± 2.98 | 0.74 ± 2.76 | -3.04 ± 3.22 | -5.04 ± 3.17 |
| Y_Sella | 0.36 ± 2.21 | 0.37 ± 2.67 | -0.76 ± 2.41 | -1.03 ± 2.29 | 0.55 ± 2.25 | 0.83 ± 2.12 |
| Y_Basion | 1.49 ± 2.72 | -1.4 ± 2.33 | -0.16 ± 4.25 | -0.86 ± 2.63 | 0.36 ± 3.46 | 0.28 ± 4.71 |
| Y_PNS | -0.02 ± 2.03 | 1.1 ± 1.91 | -0.89 ± 2.28 | -0.72 ± 2.17 | 0.39 ± 2.56 | 0.29 ± 2.62 |
| Y_A.Point | -1.35 ± 2.25 | 1.78 ± 2.42 | -1.55 ± 2.71 | -1.35 ± 2.6 | 1.42 ± 3.18 | 3.04 ± 2.89 |
| Y_B.Point | 1.97 ± 2.49 | -1.54 ± 2.65 | 1.78 ± 3.08 | -1.37 ± 2.23 | -1.32 ± 3.38 | -0.22 ± 4.13 |
| Y_Pogonion | 3.37 ± 2.27 | -0.69 ± 2.12 | 0 ± 2.2 | 0.77 ± 2.33 | -2.77 ± 2.12 | -4.01 ± 2.41 |
| Y_Menton | 2.83 ± 1.75 | -0.32 ± 1.6 | 0.23 ± 1.96 | 0.5 ± 1.94 | -2.64 ± 2.01 | -3.47 ± 2.18 |
| Y_Gonion | -4.46 ± 3.16 | -0.57 ± 3.59 | 1.52 ± 3.75 | 4.1 ± 3.4 | 1.57 ± 4.03 | -1.2 ± 4.05 |
| Y_Ramus.Point | -0.85 ± 3.87 | 0.81 ± 4.34 | -1.47 ± 3.9 | 4.48 ± 3.39 | -0.72 ± 4.13 | -4.48 ± 5.37 |
| Y_Distal.Aspect.of.Condyle | -0.86 ± 2.01 | -2.85 ± 1.89 | 3.75 ± 2.4 | -2.11 ± 1.85 | 0.21 ± 1.95 | 4.9 ± 2.34 |
| Y_Condylion | -0.96 ± 1.86 | -2.07 ± 2.27 | 2.3 ± 2.62 | -1.09 ± 2.02 | 0.67 ± 1.96 | 3.39 ± 2.42 |
| Y_Nasion | -1.52 ± 3.39 | 5.37 ± 2.69 | -4.75 ± 3.6 | -1.32 ± 3.22 | 2.29 ± 3.1 | 0.64 ± 4.06 |

Table D.3– Cephalometrics measurements per cluster (mean ± sd).

|  | Cluster 1 | Cluster 2 | Cluster 3 | Cluster 4 | Cluster 5 | Cluster 6 |
| --- | --- | --- | --- | --- | --- | --- |
| FH - SN (º) | 3 ± 3.11 | 11.19 ± 3.28 | 10.19 ± 3.71 | 10.32 ± 3.1 | 10.27 ± 3.97 | 10.46 ± 4.61 |
| SNA (º) | 80.57 ± 3.75 | 77.93 ± 3.43 | 80.19 ± 4.02 | 80.66 ± 3.44 | 78.55 ± 4.07 | 77.76 ± 4.32 |
| SNB (º) | 82.61 ± 4.16 | 78.38 ± 3.43 | 87.5 ± 3.89 | 83.29 ± 3.45 | 82.72 ± 3.38 | 84.96 ± 4.6 |
| ANB (º) | -2.04 ± 2.45 | -0.45 ± 2.43 | -7.31 ± 2.88 | -2.64 ± 2.46 | -4.17 ± 2.58 | -7.2 ± 3.4 |
| SND (º) | 80.46 ± 3.88 | 76.06 ± 3.23 | 83.98 ± 4.24 | 81.22 ± 3.52 | 80.08 ± 3.27 | 81.65 ± 4.66 |
| Y-Axis (SGn-SN) (º) | 64.43 ± 3.56 | 71.07 ± 3.25 | 62.98 ± 3.87 | 65.19 ± 3.27 | 68.7 ± 3.64 | 68.71 ± 4.8 |
| SN - GoGn (º) | 24.42 ± 4.7 | 35.39 ± 5.4 | 29.6 ± 5.59 | 32.18 ± 4.45 | 36.32 ± 5.26 | 36.85 ± 7.44 |
| Cranio-Mx Base/SN-Palatal Plane (º) | 7.91 ± 3.44 | 9.96 ± 3.65 | 7.4 ± 3.55 | 8.25 ± 3.79 | 7.71 ± 4.18 | 6.82 ± 4.03 |
| Occ Plane to SN (º) | 12.27 ± 4.35 | 18.63 ± 4.03 | 13.36 ± 4.55 | 14.4 ± 4.95 | 17.5 ± 5.23 | 16.53 ± 5.14 |
| Occ Plane to FH (º) | 2.26 ± 3.98 | 7.43 ± 3.94 | 3.17 ± 4.73 | 4.07 ± 4.79 | 7.23 ± 5.39 | 6.08 ± 5.05 |
| Facial Axis-Ricketts (NaBa-PtGn)(º) | 93.44 ± 3.74 | 85.69 ± 3.37 | 96.39 ± 4.81 | 92.67 ± 3.92 | 88.58 ± 3.75 | 90.25 ± 5.14 |
| FMA (MP-FH) (º) | 16.8 ± 4.45 | 26.72 ± 4.63 | 22.47 ± 4.89 | 24.34 ± 4.58 | 28.88 ± 5.76 | 29.83 ± 7.4 |
| Lower Face Height (ANS-Xi-Pm)(º) | 40.85 ± 3.52 | 47.68 ± 5.83 | 41.09 ± 3.41 | 43.53 ± 4.24 | 49.1 ± 4.09 | 49.03 ± 8.84 |
| Facial Angle (FH-NPo) (º) | 93.93 ± 3.19 | 90.18 ± 2.89 | 97.34 ± 4.12 | 94.59 ± 3.82 | 93.2 ± 3.82 | 94.83 ± 5.24 |
| N-A-Pg (º) | -7.09 ± 5.32 | -2.14 ± 4.88 | -13.83 ± 6.87 | -7.43 ± 5.32 | -8.61 ± 4.81 | -13.02 ± 8.36 |
| Facial Taper (º) | 69.27 ± 3.38 | 63.11 ± 3.62 | 60.2 ± 4.01 | 61.06 ± 3.38 | 57.91 ± 3.88 | 55.34 ± 5.83 |
| Gonial/Jaw Angle (Ar-Go-Me) (º) | 119.13 ± 5.94 | 126.44 ± 6.98 | 130.5 ± 6.54 | 129.3 ± 6.16 | 132.12 ± 6.23 | 136.26 ± 8.66 |
| Upper Gonial Angle (Ar-Go-Na) (º) | 49.61 ± 3.93 | 48.17 ± 4.42 | 53.74 ± 4.02 | 53.3 ± 4.25 | 49.65 ± 3.98 | 49.51 ± 3.88 |
| Lower Gonial Angle (Na-Go-Me) (º) | 69.51 ± 3.64 | 78.27 ± 4.04 | 76.75 ± 4.35 | 76 ± 3.91 | 82.47 ± 4.28 | 86.74 ± 6.55 |
| Articular Angle (º) | 141.64 ± 6.38 | 145.7 ± 7.33 | 137.97 ± 8.07 | 141.5 ± 7.22 | 144.4 ± 7.63 | 140.41 ± 5.77 |
| Saddle/Sella Angle (SN-Ar) (º) | 126.03 ± 5.14 | 125.77 ± 5.36 | 124.2 ± 6.46 | 123.86 ± 5.08 | 122.64 ± 6.31 | 123.61 ± 5.12 |
| Superior Angle (SN-AB) (º) | 85.92 ± 7.04 | 79.04 ± 5.74 | 98.3 ± 6.51 | 87.11 ± 5.75 | 88.46 ± 5.01 | 95.72 ± 12.84 |
| Rp-FH (º) | 77.67 ± 4.99 | 80.27 ± 5.54 | 71.97 ± 5.65 | 75.04 ± 5.61 | 76.76 ± 5.21 | 73.58 ± 5.98 |
| Anterior Cranial Base (SN) (mm) | 70.77 ± 10.57 | 68.15 ± 8.79 | 78.25 ± 25.85 | 68.63 ± 5.65 | 71.76 ± 17.83 | 69.2 ± 16.88 |
| Anterior Face Height (NaMe) (mm) | 119.19 ± 18.6 | 125.84 ± 16.19 | 135.64 ± 46.28 | 119.08 ± 9.59 | 136.77 ± 35.83 | 136.84 ± 32.77 |
| Upper Face Height (N-ANS) (mm) | 52.88 ± 7.68 | 54.3 ± 6.86 | 59.24 ± 20.59 | 51.82 ± 4.13 | 56.71 ± 13.78 | 55.91 ± 12.67 |
| Lower Face Height (ANS-Me) (mm) | 66.89 ± 11.68 | 72.6 ± 10.11 | 77.2 ± 26.34 | 67.69 ± 7.22 | 80.92 ± 23.09 | 81.97 ± 21.41 |
| Posterior Cranial Base (S-Ar) (mm) | 35.18 ± 5.77 | 34.8 ± 5.18 | 34.64 ± 12.83 | 32.82 ± 4.27 | 34.44 ± 9.32 | 32.05 ± 8.61 |
| Posterior Face Height (SGo) (mm) | 84.59 ± 14.22 | 79.24 ± 11.31 | 88.03 ± 31.72 | 74.53 ± 7.26 | 84.24 ± 22.28 | 85.57 ± 19.35 |
| Ramus Height (Ar-Go) (mm) | 54.26 ± 10.11 | 48.18 ± 7.88 | 59.33 ± 21.34 | 46.13 ± 5.11 | 54.04 ± 14.73 | 58.41 ± 12.41 |
| Co-Go (mm) | 69.34 ± 13.52 | 63.02 ± 9.44 | 77.54 ± 29.98 | 59.56 ± 6.24 | 69.68 ± 19.07 | 76.9 ± 17.86 |
| Convexity (A-NPo) (mm) | -3.47 ± 2.81 | -1.06 ± 2.72 | -7.76 ± 4.71 | -3.64 ± 2.64 | -4.82 ± 3.08 | -6.97 ± 3.98 |
| Maxillary Skeletal (A-Na Perp) (mm) | 0.57 ± 3.16 | -0.87 ± 3.38 | 0.53 ± 5.13 | 1.01 ± 3.45 | -1.09 ± 5.85 | -1.76 ± 6.84 |
| Midface Length (Co-A) (mm) | 87.41 ± 13.25 | 83.13 ± 11.32 | 95.64 ± 32.53 | 85.21 ± 7.45 | 87.34 ± 22.98 | 83.16 ± 20.56 |
| Ar - A (mm) | 86.99 ± 13 | 82.06 ± 11.01 | 93.39 ± 30.82 | 83.09 ± 7.05 | 84.82 ± 21.86 | 81.48 ± 19.86 |
| Maxillary length (ANS-PNS) (mm) | 53.74 ± 8.98 | 51.45 ± 6.48 | 62.9 ± 21.81 | 51.12 ± 4.59 | 55.74 ± 14.86 | 58.79 ± 15.36 |
| Pog - NB (mm) | 2.47 ± 2.65 | 1.23 ± 2.62 | -0.73 ± 3.23 | 1.94 ± 2.03 | 0.41 ± 3.57 | -1.39 ± 3.19 |
| Mand. Skeletal (Pg-Na Perp) (mm) | 7.65 ± 6.61 | 0.44 ± 6.78 | 16.62 ± 11.81 | 8.97 ± 7.55 | 7.57 ± 11.81 | 11.22 ± 14.47 |
| Mandibular Body Length (Go-Gn) (mm) | 85.67 ± 14.28 | 82.47 ± 11.36 | 98.38 ± 33.9 | 86.13 ± 7.86 | 90.89 ± 24.44 | 88.28 ± 24.02 |
| Length of Mand Base (Go-Pg) (mm) | 74.45 ± 11.48 | 72.98 ± 11.33 | 83.98 ± 27.35 | 78.47 ± 7.7 | 79.98 ± 22.81 | 71.62 ± 17.62 |
| Mandibular length (Co-Gn) (mm) | 122.92 ± 20.69 | 119.82 ± 17.1 | 145.23 ± 50.22 | 122.81 ± 10.62 | 135.58 ± 36 | 137.22 ± 31.43 |
| Co-B1 Total mand (mm) | 120.34 ± 20.23 | 117.65 ± 17.09 | 142.54 ± 49.62 | 120.44 ± 10.64 | 132.79 ± 35.73 | 133.96 ± 30.69 |
| Ar - Gn (mm) | 116.92 ± 19.38 | 112.43 ± 16.21 | 137.3 ± 46.2 | 115.31 ± 9.51 | 127.14 ± 33.19 | 129.64 ± 29.31 |
| Basal Width (mm) | 7.96 ± 2.03 | 7.04 ± 1.51 | 8 ± 3.57 | 6.81 ± 1.64 | 6.85 ± 2.57 | 7.08 ± 2.91 |
| Mx/Md diff (Co-Gn - Co-A) (mm) | 35.5 ± 8.44 | 36.69 ± 6.84 | 49.58 ± 18.6 | 37.6 ± 5.57 | 48.23 ± 13.84 | 54.07 ± 13.58 |
| Wits Appraisal (mm) | -5.4 ± 4.41 | -5.66 ± 3.98 | -16.18 ± 7.56 | -7.88 ± 4.33 | -12.66 ± 5.24 | -16.64 ± 5.13 |
| P-A Face Height(S-Go/N-Me) (%) | 70.97 ± 4.34 | 63.01 ± 4.46 | 64.76 ± 4.77 | 62.62 ± 3.89 | 61.69 ± 4.3 | 62.88 ± 5.79 |
| PFH:AFH (%) | 58.05 ± 4.2 | 50.08 ± 3.99 | 56.74 ± 4.62 | 50.05 ± 3.67 | 51 ± 4.39 | 56.48 ± 5.56 |
| S-Ar/Ar-Go (%) | 65.46 ± 7.61 | 72.83 ± 9.28 | 58.91 ± 8.77 | 71.65 ± 10.07 | 64.1 ± 7.32 | 55.01 ± 8.24 |
| UFH (N-ANS/(N-ANS+ANS-Me)) (%) | 44.2 ± 2.16 | 43.03 ± 2.12 | 43.41 ± 2.09 | 43.07 ± 2.57 | 41.59 ± 2.62 | 40.68 ± 2.42 |
| LFH/TFH (ANS-Me:N-Me) (%) | 55.88 ± 2.16 | 56.79 ± 2.22 | 56.89 ± 2.18 | 56.96 ± 2.57 | 58.48 ± 2.64 | 59.52 ± 2.47 |
| Face Ht Ratio (N-ANS/ANS-Me) (%) | 0.79 ± 0.08 | 0.76 ± 0.07 | 0.77 ± 0.08 | 0.76 ± 0.08 | 0.71 ± 0.08 | 0.69 ± 0.08 |
| SN/GoMe (%) | 101.17 ± 7.75 | 98.54 ± 8.73 | 98.01 ± 7.98 | 92.21 ± 7.68 | 94.27 ± 8.8 | 102.54 ± 10.44 |
| ANS-PNS/Me-Go (%) | 0.76 ± 0.07 | 0.75 ± 0.08 | 0.78 ± 0.09 | 0.69 ± 0.07 | 0.74 ± 0.09 | 0.85 ± 0.1 |

1. *Clustering stability and prediction accuracy of subphenotypes for new patients*


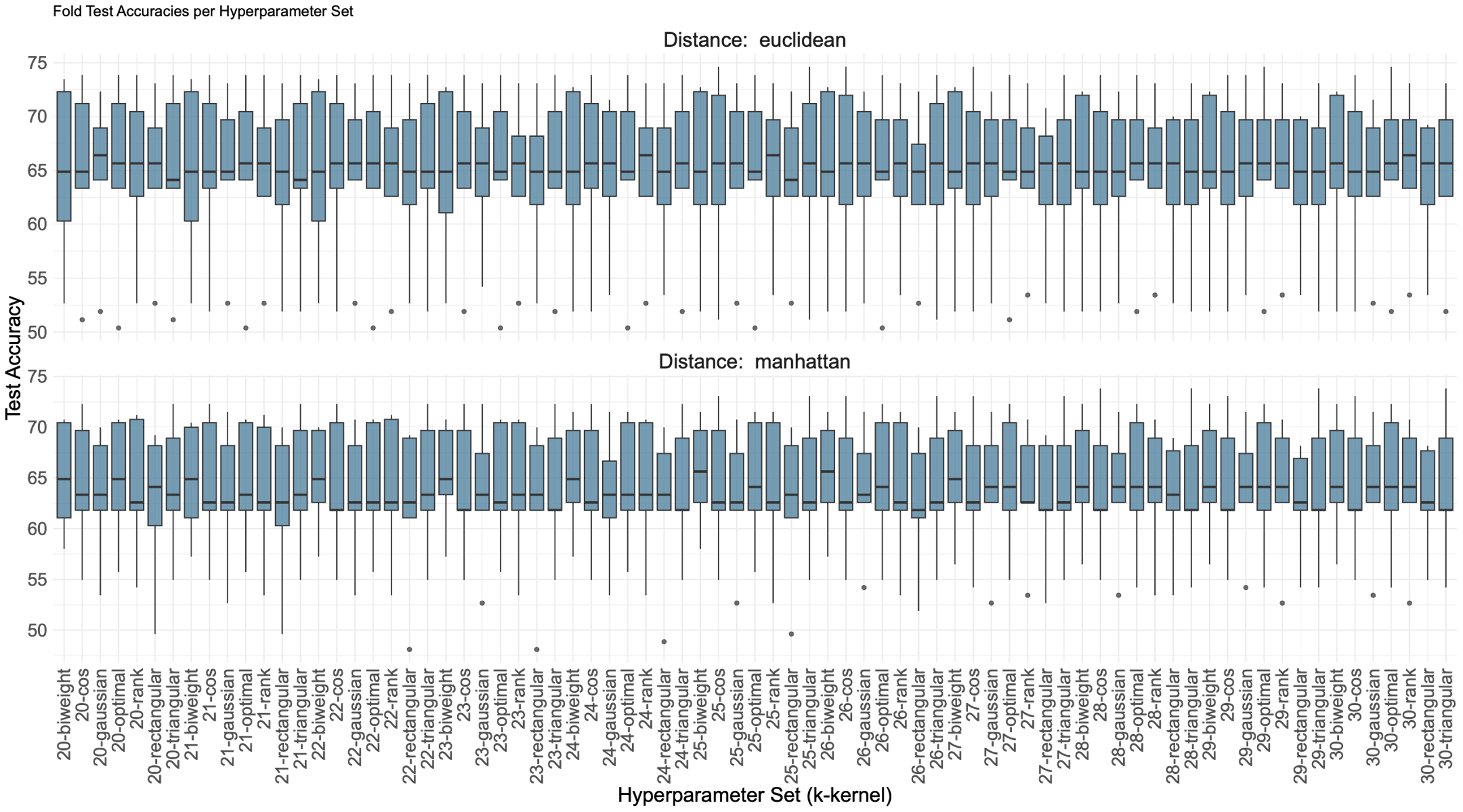


*Figure E.1 – Distribution of test dataset accuracies across the different cross-validation folds for distinct combinations of KNN hyperparameters: distance metric (top and bottom boxplots); number of nearest neighbors (k), and kernel (different boxplot). No significant differences were observed in the test accuracy by changing the different KNN hyperparameters.*


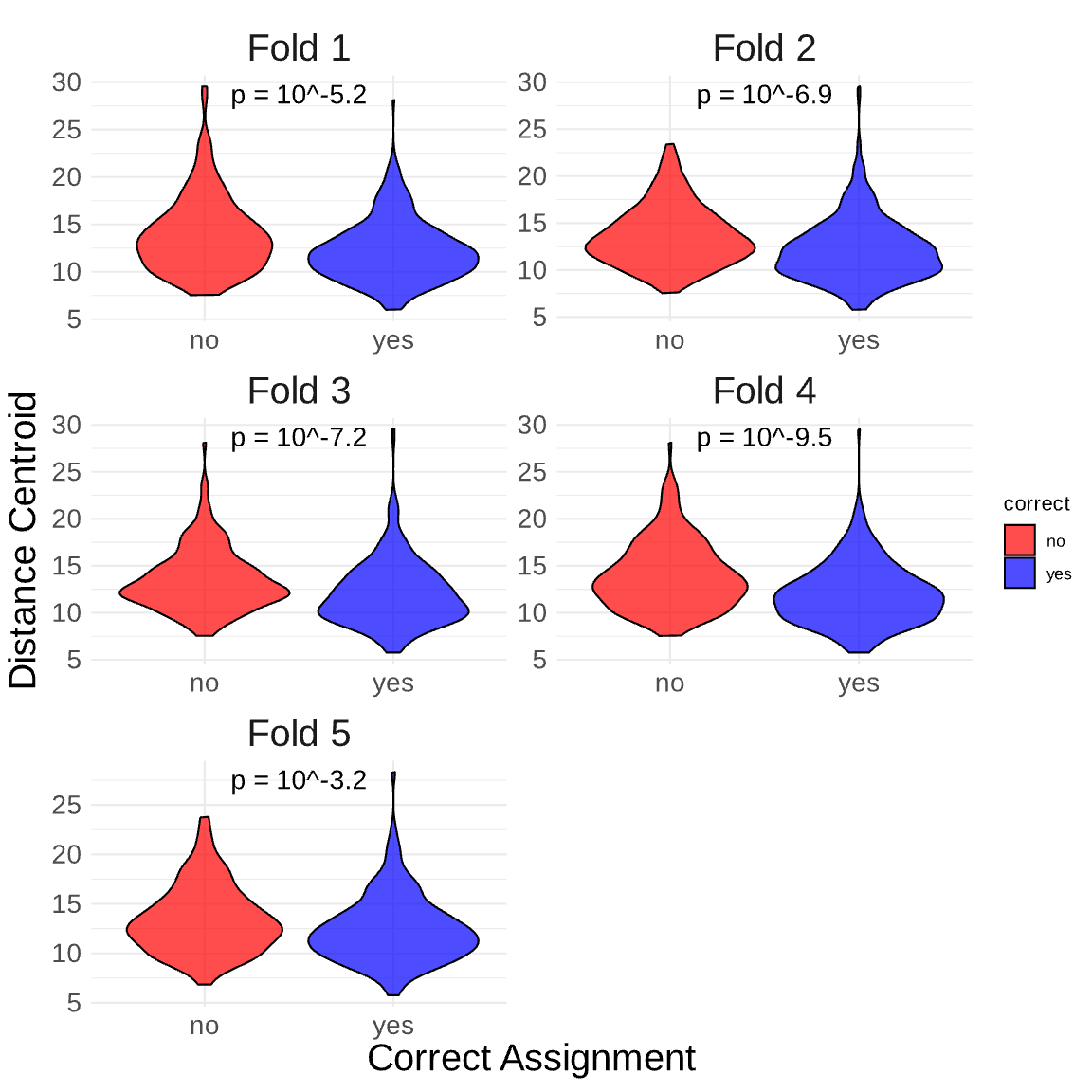


*Figure E.2 – Distribution of distances to the respective cluster centroid in the ground truth dataset for patients with either correct or incorrect cluster assignment in the different cross-validation training folds. In all folds, the p-value depicted in the figure from the Wilcoxon rank test indicates that the distance is lower for correct (blue) compared to incorrect (red) cluster assignments.*

1. *SCIII subphenotypes are generalizable to external datasets*


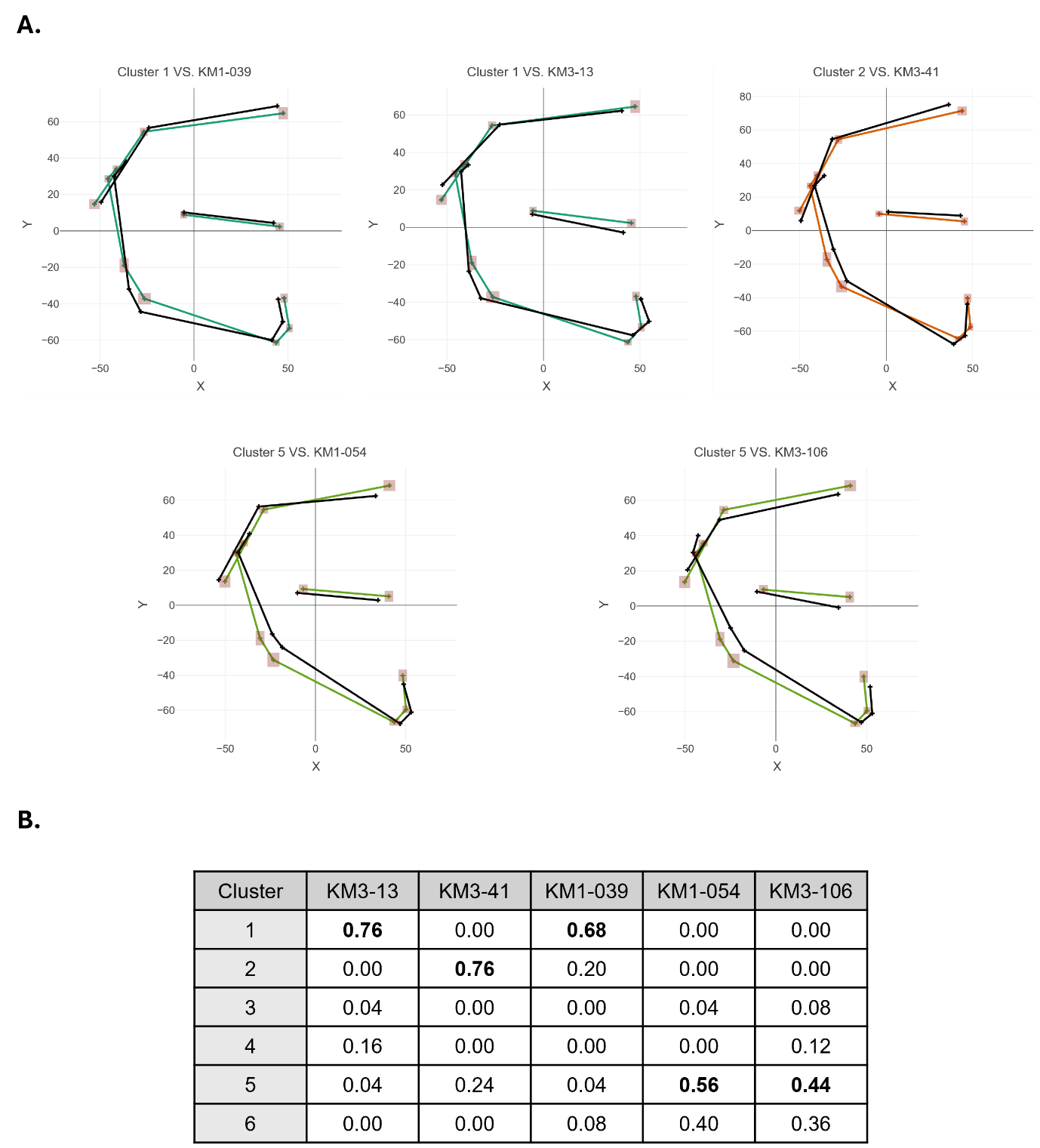


*Figure F.1 – Analysis of identified outliers in the Korean external dataset. (A) Morphological comparison of the identified outliers (black) with the mean shapes of their assigned clusters (colored). (B) Cluster membership probabilities of the identified outliers based on KNN analysis.*


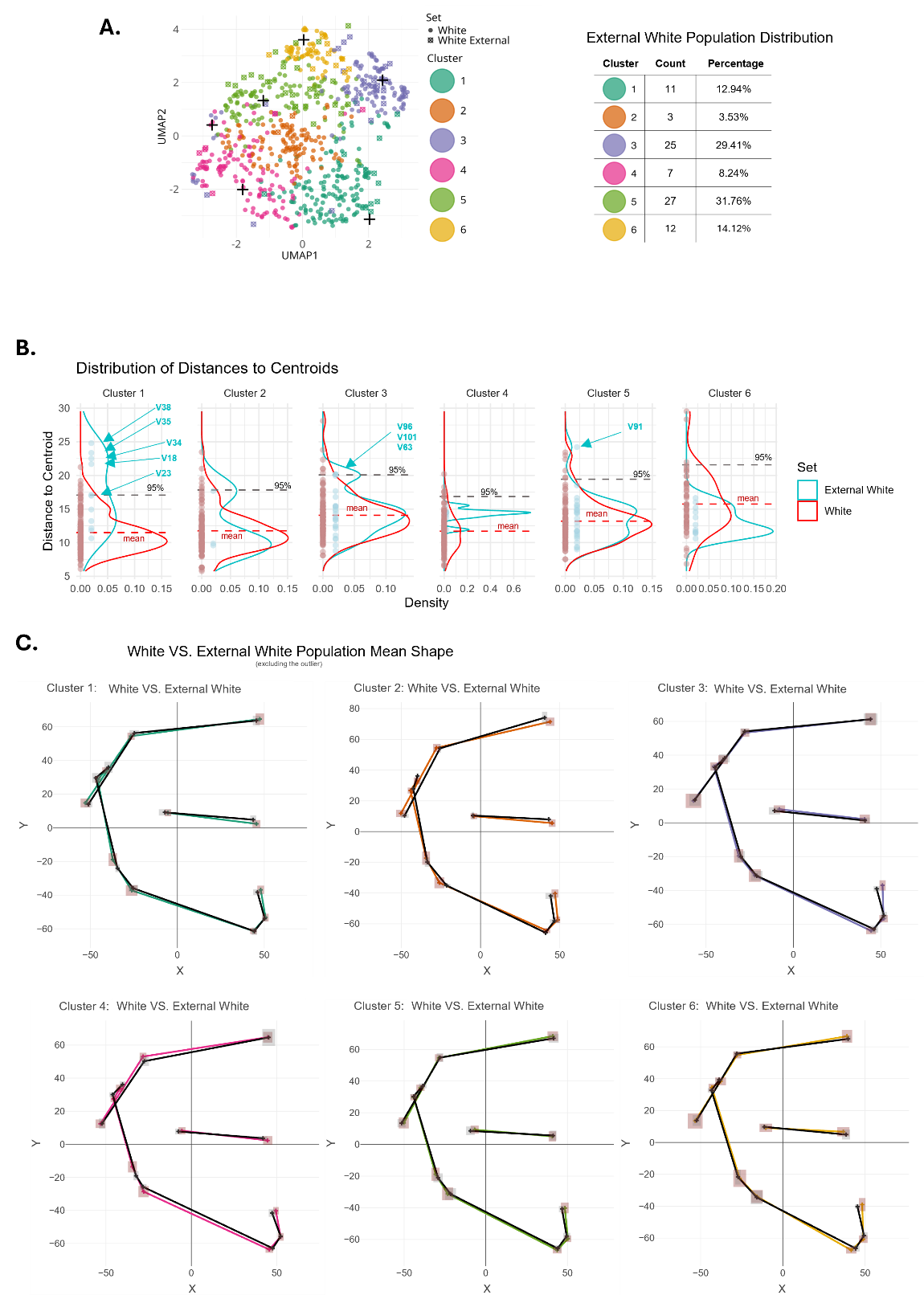


*Figure F.2 – External validation of SCIII subphenotypes - prediction in external white patients. (A) UMAP projection of both training white and external white datasets (85 patients) along with the assigned cluster/subphenotype (represented by the different colors) (left). Black crosses indicate the centroids of each cluster. Table with the distribution of patients in the external white dataset across the six predefined clusters (right). (B) Distance-to-centroid distribution analysis comparing external white (cyan) and training white (red) populations, with identification of potential outliers (exhibiting a distance larger than the 95^th^ percentile observed for the training white distribution, black dashed lines): V38, V35, V34, V18 and V23 in Cluster 1 and V96, V101; V63 in Cluster 2; and V91 in Cluster 5. (C) Morphological comparison using the mean shape of the training white patients (colored) versus the external white patients mean shape (black), by cluster.*


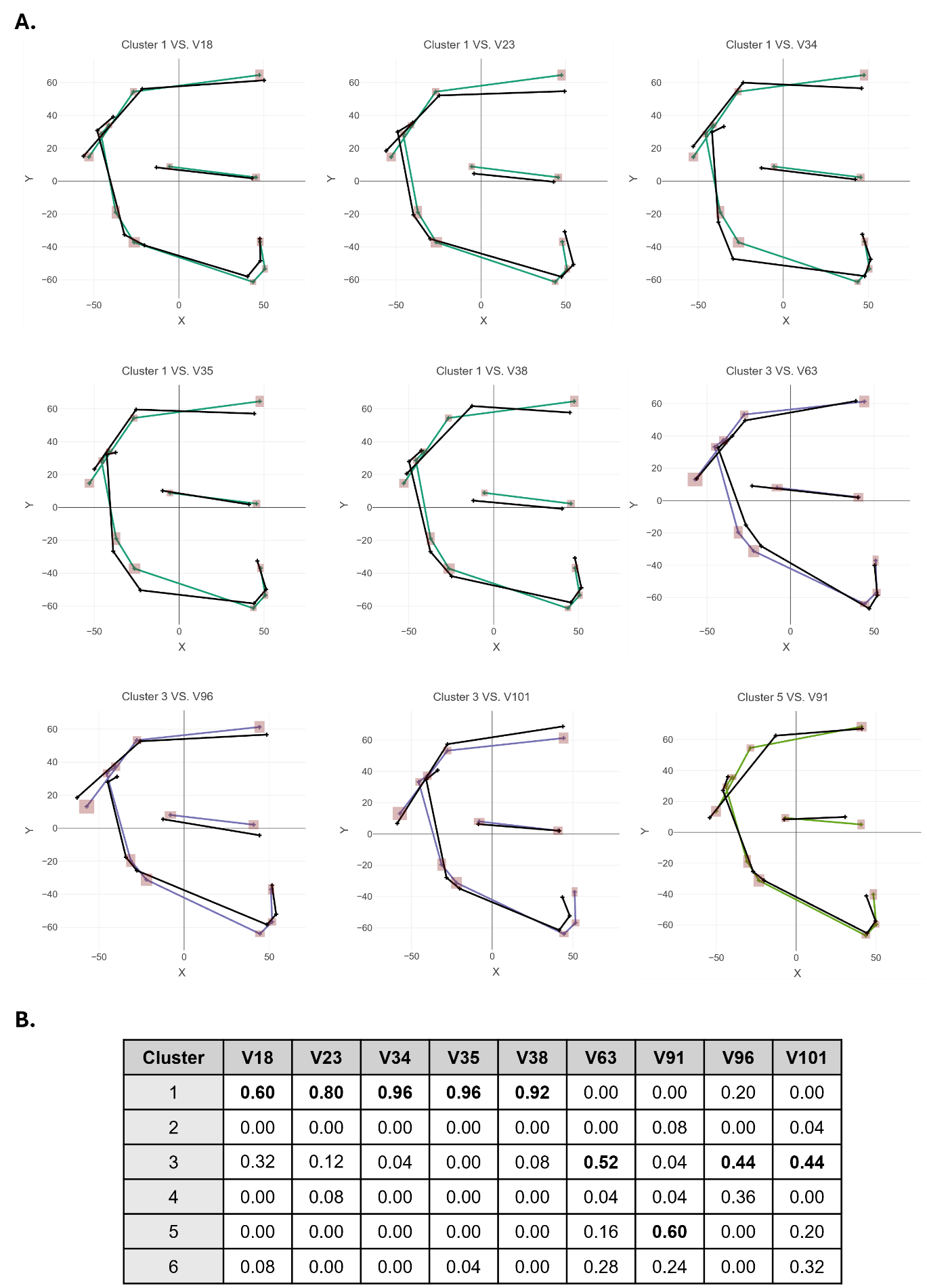


*Figure F.3 – Analysis of identified outliers in the external white dataset. (A) Morphological comparison of the identified outliers (black) with the mean shapes of their assigned clusters (colored). (B) Cluster membership probabilities of the identified outliers based on KNN analysis.*

1. *SCIII subphenotypes are associated with choice of corrective treatment.*


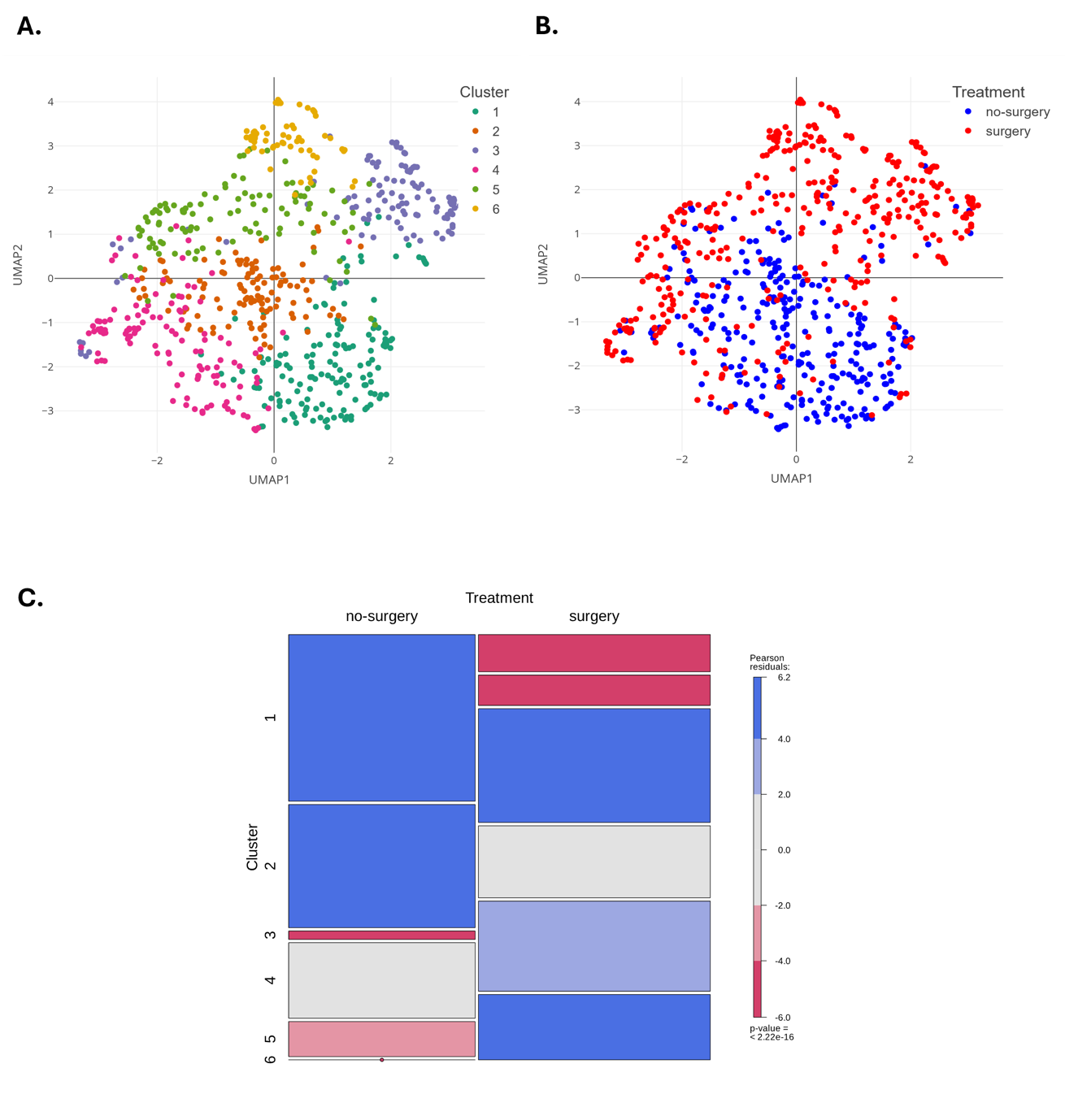


*Figure G.1 – Subphenotypes and their correlation with clinician-defined treatment. (A) UMAP representation of the patients’ Procrustes coordinates residuals and the cluster assignment (represented by different colors), each point representing a patient; (B) UMAP representation of the patients’ Procrustes coordinates residuals and the predicted treatment (represented by the different colors), each point representing a patient; (C) Mosaic plot shows a statistically significant association between the cluster identity and the treatment option (with a chi-squared p-value<0.05). Pearson residuals indicate the degree and direction of deviation from what would be expected under the assumption of independence between variables. Positive Pearson residuals indicate an observed count higher than expected (in blue), while negative Pearson residuals indicate that observed counts were lower than expected (in red).*


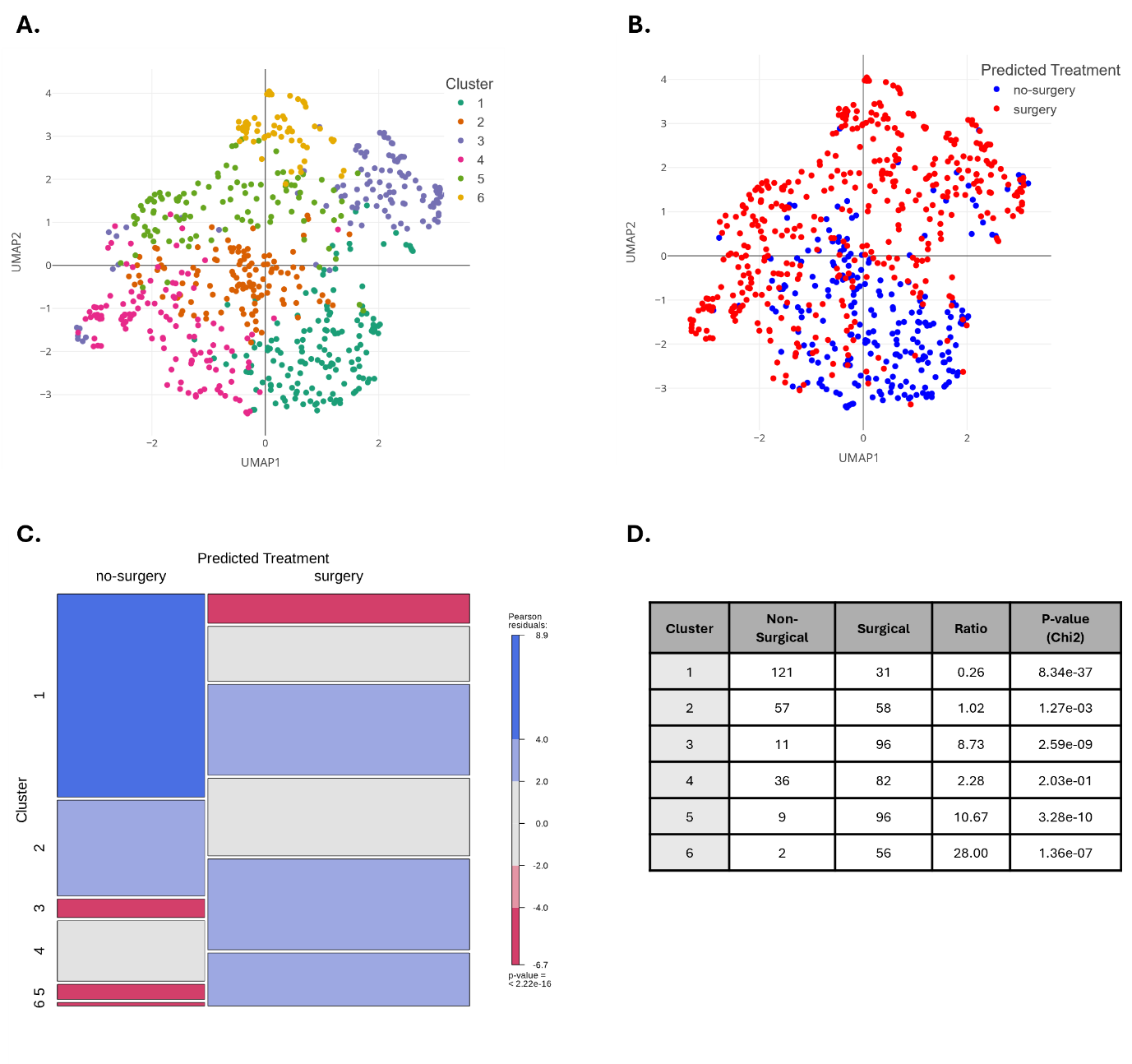
 *Figure G.2 – Subphenotypes and their correlation with predicted treatment using Stellzig-Eisenhauer et al. model. (A) UMAP representation of the Procrustes coordinates residuals and the cluster assignment (represented by the different colors), each dot representing a patient; (B) UMAP representation of the Procrustes coordinates residuals and the predicted treatment (represented by the different colors), each dot representing a patient; (C) Mosaic plot shows a statistically significant association between the cluster identity and the treatment option (with a chi-squared p-value<0.05). Pearson residuals indicate the degree and direction of deviation from what would be expected under the assumption of independence between variables. Positive Pearson residuals indicate an observed count higher than expected (in blue), while negative Pearson residuals indicate that observed counts were lower than expected (in red). (D) Distribution of Non-surgical and Surgical per cluster according to the Stellzig-Eisenhauer. et al. formula, along with the respective significance test based on chi-squared comparing the observed and the expected number of patients across the two treatment options.*

1. *SCIII subphenotypes diagnostic tool*


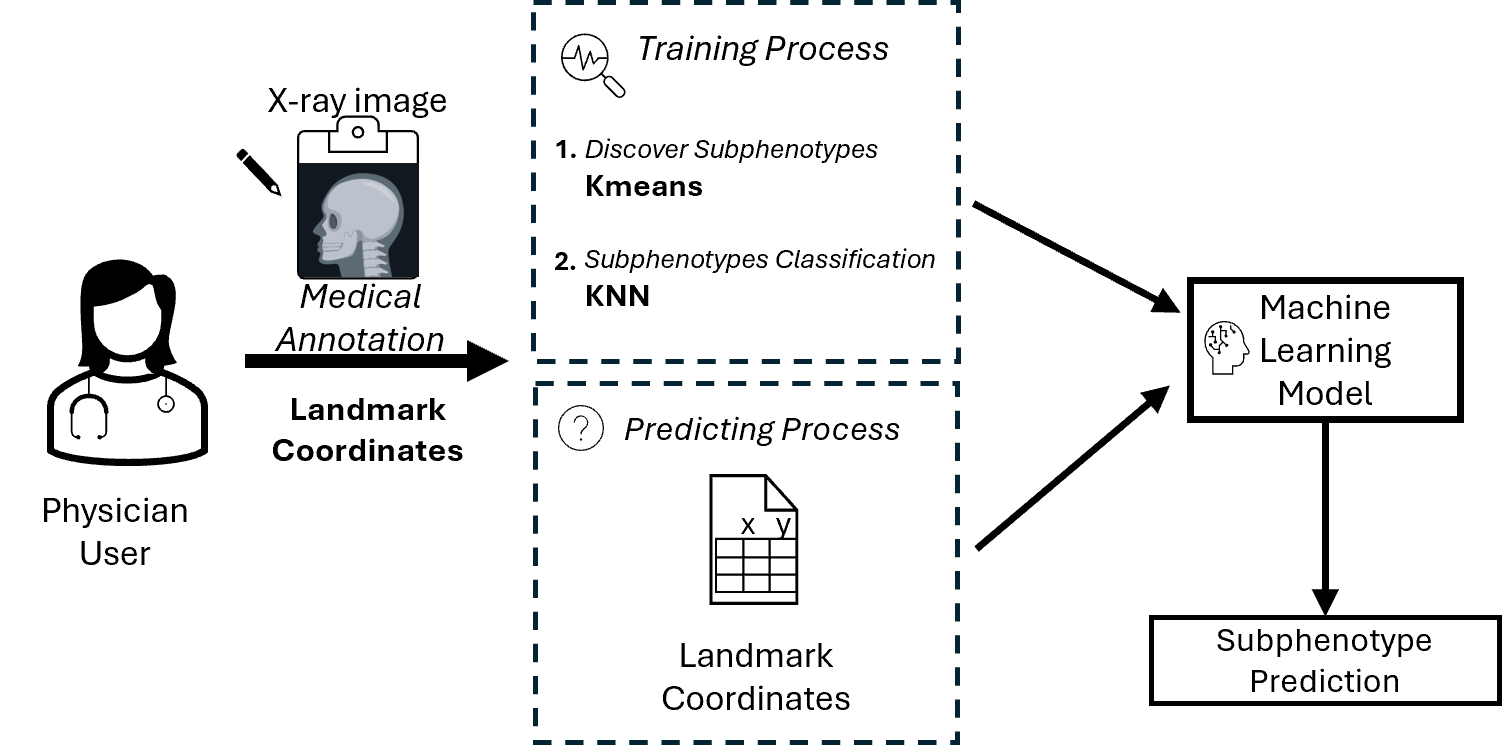


*Figure H – Summary of the workflow of our Geometric morphometrics based diagnostic prediction model for Skeletal Class III patients. After the training process, the clinician can interact with the model by first annotating the landmarks of a lateral cephalogram x-ray and then predicting the respective patient subphenotype. This tool is freely available at* [*https://tools.istars.pt/sciii/*](https://tools.istars.pt/sciii/) *.*
