# Supplementary material for: Geometric morphometrics based diagnostic model for Skeletal Class III patients
Source: Commun Med (Lond). 2026 Apr 14;6:340. doi: 10.1038/s43856-026-01557-y (PMC13270103; doi:10.1038/s43856-026-01557-y)
Supplement: Supplementary file 1 — Transparent Peer Review file [file 43856_2026_1557_MOESM1_ESM.pdf]

nature portfolio

Peer Review File

Geometric morphometrics based diagnostic model for Skeletal Class III patients

Corresponding Author: Professor Joao Guimaraes

This file contains all reviewer reports in order by version, followed by all author rebuttals in order by version.

Version 0:

Reviewer comments:

Reviewer #1

(Remarks to the Author)
The article presents a ML-based approach for classification of Class III individuals. Unless I see the algorithm working (running it), I am not sure I can assess the correctness of it, but assuming it is all ok, I think proposing new, cephalometrics-free approaches is always a good contribution to the field.
My main concerns have to do with what the main parts of the article say they do, and what the authors actually do (or at least, how it is presented). For example, it is said (even in the title) that there is an aim of “predicting”. But it is very late explained what the prediction is about (type of treatment) and then, very little elaborated about it (validation of the indication, a discussion about it). It seems more like what is being done is indeed a classification, which can eventually lead to a prediction model of treatment type needed (which should be discussed). A thorough classification of skeletal malocclusions using geometric morphometrics is itself a good aim, although it has been attempted for decades now. So, the ML approach does give an extra.
I believe if the focus of the article is clearly stated and written in function of it, many things will fall in place.
Minor comments:
- Please explain in more detail how was observer error assessed. An ANOVA is mentioned, but not detailed what data was used, whether a Procrustes approach was used (the table in the sup. File shows twice “landmarks” as effect, why?). The same goes for the ICC calculation.
- Since much of the readership will be people at least a bit familiar with geometric morphometrics, I suggest to use more familiar ways to present parts of the information. For example: Fig 1C and 1D are not standard ways to show GMM-based results (A table of explained variance suffices. Eigenvalues are not normally used).
- The text with the results describing the cluster features is very long and hard to follow. I suggest to use Figure 3 to reproduce what is being described, using visual cues such as arrows showing “displacement” etc. Do you have a way to “amplify” the features to see them better in the figure?

Reviewer #2

(Remarks to the Author)
Areas for Improvement
1. Missing methodological details early in the paper. The Methods section is located at the end, which impairs readability and comprehension in the early stages. The GPA, clustering steps, and KNN model should be summarized earlier. Please put your methodology before the result.
2. Terms like "Procrustes residuals" or "cluster centroid distance" could be better explained for readers from non-statistical backgrounds. Definitions or schematic figures would aid understanding.
3. Limited External Validation (n=96 Korean patients). While the Korean dataset provides useful cross-ethnic insight, its limited size (especially with some clusters like C3 and C6 underrepresented) may limit generalizability. The authors acknowledge this, but further multicenter external validation is necessary.
4. Treatment assignment was subjective. The classification into surgical vs. non-surgical groups is based on two orthodontists’ opinions. While both are experienced, this could introduce bias. An objective scoring system (e.g., integrating cephalometric thresholds) could complement the clinician-based grouping.
5. Lack of Open Access Code and Model. The manuscript mentions that code and data will be made available post-publication. However, reproducibility is a core scientific value. Pre-acceptance availability (e.g., on GitHub or Zenodo) would

enhance transparency and replicability.

Suggestions for Enhancement

• Consider integrating a user-friendly web interface for the diagnostic model in future work, particularly one that allows uploading landmark data and returns a cluster classification and treatment recommendation.
• Conduct longitudinal follow-up to determine whether treatment decisions based on cluster classification yield better functional or aesthetic outcomes.
• Include a decision-tree or flowchart summarizing the clustering process and treatment mapping to guide clinicians.

Conclusion

This article presents an important contribution to the orthodontic field by leveraging advanced morphometric and computational techniques for SCIII classification. It not only improves upon existing phenotype stratification but also introduces a scalable and generalizable model for precision orthodontics. Minor limitations—mainly in the external validation cohort size and subjective treatment classification—do not diminish the overall impact and potential of the work.

Reviewer #3

(Remarks to the Author)
The manuscript reports using 655 adult SCIII cases for clustering without any sample size or power calculation to justify that cohort size is sufficient for PCA, k-means or chi-square tests

No explicit criterion for premolar extraction versus entire-arch distalization is given, yet clinical examples show both approaches: Son et al. achieved presurgical decompensation via entire upper-arch distalization without premolar extraction (DOI: 10.25259/APOS_78_2023), while Nguyen et al. extracted third molars to permit lower-arch distalization (DOI: 10.1097/MD.0000000000040832). These references should also be cited.

The study does not differentiate true skeletal Class III from pseudo-Class III (functional shift): although clustering is shape-driven, no clinical “dual-bite” or crossbite assessment was used to exclude functional mandible shifts

Incisor inclination landmarks are omitted: upper-incisor proclination (U1-SN) and lower-incisor retroclination (IMPA) correlate with dental compensation severity and surgical candidacy but were not included in the 12-landmark set

Recommendation: Major Revision

Version 1:

Reviewer comments:

Reviewer #1

(Remarks to the Author)
I appreciate the changes/responses given by the authors, particularly the possibility to test the model through the website provided.
I only have a comment on error, and a couple minor concerns of editorial nature:
1) How sensitive can be the model to observer error? Some ICCs were below 0.5 even. I understand that one or two landmarks that are prone to error may not impact the model, what about more of them, or lower ICCs. Some comment about it in the discussion would be appropriate.
2) Please double-check magnitudes and units (they should be consistently spaced)
3) I did not find in the text the language/library used to build the code (unless I missed it). R was mentioned but I understood that it was only for statistical analyses.

Reviewer #2

(Remarks to the Author)
Authors have addressed all my comments. Please add DOI to References 1, 30, 31, 37, 42.

Reviewer #3

(Remarks to the Author)
I dont see any response and revision according to reviewer 3 comments.

Version 2:

Reviewer comments:

Reviewer #3

(Remarks to the Author)
The authors have thoroughly revised the manuscript. I recommend acceptance in the present form.

Open Access This Peer Review File is licensed under a Creative Commons Attribution 4.0 International License, which permits use, sharing, adaptation, distribution and reproduction in any medium or format, as long as you give appropriate credit to the original author(s) and the source, provide a link to the Creative Commons license, and indicate if changes were made.
In cases where reviewers are anonymous, credit should be given to 'Anonymous Referee' and the source.
The images or other third party material in this Peer Review File are included in the article's Creative Commons license, unless indicated otherwise in a credit line to the material. If material is not included in the article's Creative Commons license and your intended use is not permitted by statutory regulation or exceeds the permitted use, you will need to obtain permission directly from the copyright holder.
To view a copy of this license, visit https://creativecommons.org/licenses/by/4.0/

Dear Reviewers,

We are pleased to submit our revised version of the manuscript entitled: *"Geometric morphometrics based diagnostic model for Skeletal Class III patients"*, following the suggestions by Reviewer #1 and Review #2. We appreciate the time spent reviewing our manuscript and the comments provided, which have been thoroughly addressed, and the manuscript has been modified accordingly. Overall, the reviewing process has helped us to further improve the quality of the paper. Please find below the point-by-point response to Reviewers.

Reviewer #1:

1."The article presents a ML-based approach for classification of Class III individuals. Unless I see the algorithm working (running it), I am not sure I can assess the correctness of it, but assuming it is all ok, I think proposing new, cephalometrics-free approaches is always a good contribution to the field.

My main concerns have to do with what the main parts of the article say they do, and what the authors actually do (or at least, how it is presented). For example, it is said (even in the title) that there is an aim of "predicting". But it is very late explained what the prediction is about (type of treatment) and then, very little elaborated about it (validation of the indication, a discussion about it). It seems more like what is being done is indeed a classification, which can eventually lead to a prediction model of treatment type needed (which should be discussed). A thorough classification of skeletal malocclusions using geometric morphometrics is itself a good aim, although it has been attempted for decades now. So, the ML approach does give an extra. I believe if the focus of the article is clearly stated and written in function of it, many things will fall in place.

We acknowledge the Reviewer #1's comment and would like to clarify that by using the term "prediction" in our study we meant to identify/classify the SCIII subphenotypes in new patients based on their skeletal features. We believe that this subphenotype classification represents a critical first step toward evidence-based clinical decision-

making. Following this reviewer's suggestion, we have revised and rephrased the manuscript to make it clearer that our study focuses on subphenotype classification.

Together with this revised version, we provide the source code (as a zip file) for the developed model, as well as for all the analysis performed to generate the figures and tables included in the manuscript. This code will become publicly available after paper publication. Additionally, we have built an interactive web app to use our model that the reviewers can accessed at: https://tools.istars.pt/sciii/

Minor comments:

1. "Please explain in more detail how was observer error assessed. An ANOVA is mentioned, but not detailed what data was used, whether a Procrustes approach was used (the table in the sup. File shows twice "landmarks" as effect, why?). The same goes for the ICC calculation."

Following the Reviewer #1's suggestion, the methods employed regarding the observer error assessment were further developed and clarified in the "reproducibility and reliability of landmark annotation" subsection of Methods (page 6). Briefly, the same 10 patients were independently measured by two operators, and the collected annotation were subject to a Generalized Procrustes Analysis (GPA), followed by the ANOVA and ICC analyses. Based on the ANOVA results, we now include separate rows for the factors Landmark, Operator, Landmark:Operator interaction, and residuals (Supplementary Table B.3).

2. "Since much of the readership will be people at least a bit familiar with geometric morphometrics, I suggest to use more familiar ways to present parts of the information. For example: Fig 1C and 1D are not standard ways to show GMM-based results (A table of explained variance suffices. Eigenvalues are not normally used)."

Thank you for the comment. To improve Figure 1 readability, we followed the Reviewer #1's suggestion and re-designed it to include a summary table of the principal component analysis (page 10). This table was previously presented in the supplementary material as Supplementary Table C.3, which has now been removed.

3. *"The text with the results describing the cluster features is very long and hard to follow. I suggest to use Figure 3 to reproduce what is being described, using visual cues such as arrows showing "displacement" etc. Do you have a way to "amplify" the features to see them better in the figure?"*

We thank Reviewer #1 for this insightful comment. In response, we have revised the Results section to provide a clearer and more concise summary of the key characteristics of each cluster (pages 12–14). Additionally, Figure 3 has been updated with new visual cues to enhance the interpretability and clarity of the cluster-specific features.

<u>Reviewer #2:</u>

Areas for improvement

1. "Missing methodological details early in the paper. The Methods section is located at the end, which impairs readability and comprehension in the early stages. The GPA, clustering steps, and KNN model should be summarized earlier. Please put your methodology before the result."

Following Reviewer #2's suggestion, we moved the methods section before the Results allowing the description of the GPA, clustering steps, and KNN to be summarized earlier in the manuscript, thereby increasing readability.

2. "Terms like "Procrustes residuals" or "cluster centroid distance" could be better explained for readers from non-statistical backgrounds. Definitions or schematic figures would aid understanding."

We acknowledge the Reviewer #2 comment and provide a summarized definition of Procrustes residuals in the "Landmark Procrustes residuals" subsection in the Methods, as well as include novel visual aids in Figure 1 to improve readability and interpretation. We have also included a new subsection "Generalizability of SCIII subphenotypes across external datasets" in the Methods (page 7) to explain the calculation of cluster centroid distances and how to interpret them.

3. "Limited External Validation (n=96 Korean patients). While the Korean dataset provides useful cross-ethnic insight, its limited size (especially with some clusters like C3 and C6 underrepresented) may limit generalizability. The authors acknowledge this, but further multicenter external validation is necessary"

Thank you for the comment. We acknowledge that the Korean dataset provides valuable cross-ethnic insights. Nevertheless, its limited size—particularly the potential underrepresentation of particular clusters such as C3 and C6—may restrict the generalizability of our findings. Therefore, to mitigate this limitation, we followed Reviewer #2's comment and we have incorporated a second external Caucasian cohort (n=85) and expanded the Korean external cohort from n=96 to n=186 patients. Important and very interesting, this external additional sample incorporation allowed us to observe that increasing the Korean cohort led to a cluster distribution consistent with the previous findings (pages 15–16). Furthermore, the additional Caucasian cohort showed us that all subphenotypes were adequately represented, supporting the robustness and generalizability of our phenotype classification to external datasets. The latter analysis has been included in the main text (page 17) and in the new Supplementary Figure F.2.

4. "Treatment assignment was subjective. The classification into surgical vs. non-surgical groups is based on two orthodontists' opinions. While both are experienced, this could introduce bias. An objective scoring system (e.g., integrating cephalometric thresholds) could complement the clinician-based grouping."

We acknowledge Reviewer #2's suggestion and included the current gold-standard formula for SCIII treatment prediction in adult patients (surgical versus non-surgical treatment based on a scoring system from *Stellzig-Eisenhauer A. et al., 2002*). Using this alternative method, we observed equivalent relationships between the identified clusters and treatment decision (page 17), which further strengths the clinical relevance of the novel SCIII subphenotypes identified in our study. This update required changes in the Methods, Results, and Supplementary Materials sections. Specifically, we created a new figure, Supplementary Figure G.2, to illustrate these findings.

5. "Lack of Open Access Code and Model. The manuscript mentions that code and data will be made available post-publication. However, reproducibility is a core scientific value. Pre-acceptance availability (e.g., on GitHub or Zenodo) would enhance transparency and replicability."

We thank Reviewer #2 for this comment. Together with this revised version, we provide the source code (as a zip file) for the developed model, as well as for all the analysis performed to generate the figures and tables included in the manuscript. This code will become publicly available after paper publication.

Suggestions for Enhancement

"Consider integrating a user-friendly web interface for the diagnostic model in future work, particularly one that allows uploading landmark data and returns a cluster classification and treatment recommendation."

We thank Reviewer #2 for this suggestion. We have developed a user-friendly web interface that allows uploading landmark data and returns cluster classification, which is already available at: https://tools.istars.pt/sciii/

"Conduct longitudinal follow-up to determine whether treatment decisions based on cluster classification yield better functional or aesthetic outcomes."

We agree that conducting a follow-up study to assess the impact of clinical decisions based on the use of the cluster classification would further enrich the current findings. For the present dataset, we do not have access to information regarding functional or aesthetic outcomes for patients who may have undergone any form of treatment.

"Include a decision-tree or flowchart summarizing the clustering process and treatment mapping to guide clinicians."

We appreciate the comment. Therefore, we included a figure as supplementary material (Supplementary Material H) illustrating the steps of model training and deployment. For the latter, the physician, as the user, annotates the anatomical landmarks on the lateral X-ray and inputs them into the model, which then outputs the patient subphenotype.

Conclusion

"This article presents an important contribution to the orthodontic field by leveraging advanced morphometric and computational techniques for SCIII classification. It not only improves upon existing phenotype stratification but also introduces a scalable and generalizable model for precision orthodontics. Minor limitations—mainly in the external validation cohort size and subjective treatment classification—do not diminish the overall impact and potential of the work."

We greatly appreciate the time and effort this referee took to evaluate our work and provide detailed feedback. We are grateful for the constructive comments and completely agree with the reviewer respecting the impact of the current paper.

Regarding the external validation cohort size, the authors acknowledge that further multicenter external validation was necessary. To overcome this limitation, the authors introduced a second Caucasian external cohort (n=85) and increased the Korean external cohort to n=186. The authors observed that by increasing the Korean external cohort a similar distribution of the population was observed in the defined clusters (page 16). The Caucasian external cohort further showed that the subphenotype classification was generalizable since all phenotypes were adequately represented in this external dataset (page 17).

Dear Reviewers,

We are pleased to submit our revised version of the manuscript entitled: *"Geometric morphometrics based diagnostic model for Skeletal Class III patients"*, following the suggestions by the three reviewers. We appreciate the time spent reviewing our manuscript and the comments provided, which have been thoroughly addressed, and the manuscript modified accordingly. Please find below the point-by-point response to Reviewers.

Reviewer #1

1) How sensitive can be the model to observer error? Some ICCs were below 0.5 even. I understand that one or two landmarks that are prone to error may not impact the model, what about more of them, or lower ICCs. Some comment about it in the discussion would be appropriate.

We would like to clarify that the ICCs presented in the supplementary data correspond to the same ten patients that were annotated by two different operators after applying our preprocessing steps, specifically the Generalized Procrustes Analysis (GPA). Importantly, we have confirmed that ICCs calculated before performing the GPA step were greater than 0.80 for all annotated landmarks, further highlighting the importance of performing adequate preprocessing steps. Nonetheless, the question raised by this reviewer is very pertinent and we further investigated their main concern regarding the sensitivity of the model. Notably, despite the presence of some landmarks prone to observer error, there was a very good agreement between the patient subphenotypes assignment probabilities across the two operators, with an average and median cosine similarity of 0.8209 and 0.9660, respectively. As suggested by the reviewer, these results and comments were added to the discussion section.

2) Please double-check magnitudes and units (they should be consistently spaced)

We thank the reviewer for raising this issue, we have now corrected the different measurements throughout the manuscript.

3) I did not find in the text the language/library used to build the code (unless I missed it). R was mentioned but I understood that it was only for statistical analyses.

Thank you for this comment. We have now clarified the programming language, software, and libraries used, and have included this information in the Methods section.

Dataset was created using Dolphin Imaging software (version 11.2; Dolphin Imaging & Management Solutions, Chatsworth, CA, USA). All analyses were conducted using R (version 4.4.0; 2024-04-24), and the following packages were used:

- psych: intraclass correlation coefficient (ICC) analysis
- shapes: Generalized Procrustes Analysis (GPA) and Principal Component Analysis (PCA)
- factoextra: k-means clustering
- kknn: k-nearest neighbors (KNN) classification

In addition, when providing the source code, we will include the corresponding .yaml file for the proper creation of a reproducible Conda environment, listing all software dependencies and package versions to ensure full reproducibility of the analyses.

Reviewer #2

1) *Authors have addressed all my comments. Please add DOI to References 1, 30, 31, 37, 42.*

As requested, DOIs have been added to the requested references in the revised manuscript.

Reviewer #3

1) The manuscript reports using 655 adult SCIII cases for clustering without any sample size or power calculation to justify that cohort size is sufficient for PCA, k-means or chi-square tests

We thank the reviewer for this comment. We would like to clarify that PCA method was applied to explore the structure of the data rather than to reduce features to be further used in the unsupervised learning step.

To ensure adequate statistical power, a sample of 655 patients was utilized alongside 24 variables (i.e. the 12 annotated landmarks in 2D space). The resulting ratio of ~27 subjects per variable falls well within the widely accepted thresholds required for robust multivariate analysis.

Our aim was to further define subphenotypes for the SCIII condition, generating clusters of patients with distinguishable features between them. To assess cluster characteristics, we used both categorical (e.g., gender, treatment) and continuous variables (e.g., cephalometric measurements and landmark coordinates). To ensure adequate cluster sizes, we performed power calculations using the “pwr” library in R:

- For continuous variables: Since these require a statistical test comparing a grouping variable across a continuous outcome, we used Cohen's f as the effect size. Assuming a moderate effect size (f = 0.25), power = 0.80, significance level = 0.05, and 6 clusters, the minimum cluster size was estimated as ~35 subjects (pwr.anova.test).
- For categorical variables: For variables such as gender and treatment (two options each), using Cohen's w for a medium effect size (w = 0.25), power = 0.80, α = 0.05, and 6 clusters (df = 5), the total sample size required was ~205, corresponding to a minimum cluster size of ~34 subjects (pwr.chisq.test).

Power calculations utilizing the negative binomial distribution indicated that 625 subjects were required to guarantee, with 95% confidence, a minimum representation of 35 subjects within each of the six different clusters. This calculation conservatively assumes a minimum subphenotype prevalence of 10% within the SCIII cohort. As our dataset (N=655) exceeds this threshold, the sample provides sufficient statistical power to stratify the cohort into six clusters and perform robust comparative analyses of continuous and categorical variables.

2) No explicit criterion for premolar extraction versus entire-arch distalization is given, yet clinical examples show both approaches: Son et al. achieved presurgical decompensation via entire upper-arch distalization without premolar extraction (DOI: 10.25259/APOS_78_2023), while Nguyen et al. extracted third molars to permit lower-arch distalization (DOI: 10.1097/MD.0000000000040832). These references should also be cited.

We thank the reviewer for this suggestion; we have added these references to the manuscript's introduction.

3) The study does not differentiate true skeletal Class III from pseudo-Class III (functional shift): although clustering is shape-driven, no clinical "dual-bite" or crossbite assessment was used to exclude functional mandible shifts

Clinical examination in centric relation was performed for all subjects and the eligibility criteria were defined to include only true skeletal Class III cases; therefore, patients with pseudo–Class III (functional shift) were excluded from the study. We appreciate this comment and have added this clarification to the Methods section.

4) Incisor inclination landmarks are omitted: upper-incisor proclination (U1-SN) and lower-incisor retroclination (IMPA) correlate with dental compensation severity and surgical candidacy but were not included in the 12-landmark set

We appreciate this comment by the reviewer; however, only exclusive skeletal landmarks were intentionally included in the model (an approach equivalent to that reported in DOI: 10.1177/0022034515581643), and dental parameters such as U1–SN and IMPA were therefore omitted.
